# Ancient DNA Reveals Prehistoric Gene-Flow from Siberia in the Complex Human Population History of North East Europe

**DOI:** 10.1371/journal.pgen.1003296

**Published:** 2013-02-14

**Authors:** Clio Der Sarkissian, Oleg Balanovsky, Guido Brandt, Valery Khartanovich, Alexandra Buzhilova, Sergey Koshel, Valery Zaporozhchenko, Detlef Gronenborn, Vyacheslav Moiseyev, Eugen Kolpakov, Vladimir Shumkin, Kurt W. Alt, Elena Balanovska, Alan Cooper, Wolfgang Haak, the Genographic Consortium

**Affiliations:** 1 Australian Centre for Ancient DNA, School of Earth and Environmental Sciences, University of Adelaide, Adelaide, South Australia, Australia; 2 Research Centre for Medical Genetics, Russian Academy of Medical Sciences, Moscow, Russia; 3 Vavilov Institute for General Genetics, Russian Academy of Sciences, Moscow, Russia; 4 Institute of Anthropology, Johannes Gutenberg University of Mainz, Mainz, Germany; 5 Kunstkamera Museum, St. Petersburg, Russia; 6 Institute for Archaeology, Russian Academy of Sciences, Moscow, Russia; 7 Faculty of Geography, Moscow State University, Moscow, Russia; 8 Römisch-Germanisches Zentralmuseum, Mainz, Germany; 9 Institute for the History of Material Culture, Russian Academy of Science, St. Petersburg, Russia; Vanderbilt University, United States of America

## Abstract

North East Europe harbors a high diversity of cultures and languages, suggesting a complex genetic history. Archaeological, anthropological, and genetic research has revealed a series of influences from Western and Eastern Eurasia in the past. While genetic data from modern-day populations is commonly used to make inferences about their origins and past migrations, ancient DNA provides a powerful test of such hypotheses by giving a snapshot of the past genetic diversity. In order to better understand the dynamics that have shaped the gene pool of North East Europeans, we generated and analyzed 34 mitochondrial genotypes from the skeletal remains of three archaeological sites in northwest Russia. These sites were dated to the Mesolithic and the Early Metal Age (7,500 and 3,500 uncalibrated years Before Present). We applied a suite of population genetic analyses (principal component analysis, genetic distance mapping, haplotype sharing analyses) and compared past demographic models through coalescent simulations using Bayesian Serial SimCoal and Approximate Bayesian Computation. Comparisons of genetic data from ancient and modern-day populations revealed significant changes in the mitochondrial makeup of North East Europeans through time. Mesolithic foragers showed high frequencies and diversity of haplogroups U (U2e, U4, U5a), a pattern observed previously in European hunter-gatherers from Iberia to Scandinavia. In contrast, the presence of mitochondrial DNA haplogroups C, D, and Z in Early Metal Age individuals suggested discontinuity with Mesolithic hunter-gatherers and genetic influx from central/eastern Siberia. We identified remarkable genetic dissimilarities between prehistoric and modern-day North East Europeans/Saami, which suggests an important role of post-Mesolithic migrations from Western Europe and subsequent population replacement/extinctions. This work demonstrates how ancient DNA can improve our understanding of human population movements across Eurasia. It contributes to the description of the spatio-temporal distribution of mitochondrial diversity and will be of significance for future reconstructions of the history of Europeans.

## Author Summary

The history of human populations can be retraced by studying the archaeological and anthropological record, but also by examining the current distribution of genetic markers, such as the maternally inherited mitochondrial DNA. Ancient DNA research allows the retrieval of DNA from ancient skeletal remains and contributes to the reconstruction of the human population history through the comparison of ancient and present-day genetic data. Here, we analysed the mitochondrial DNA of prehistoric remains from archaeological sites dated to 7,500 and 3,500 years Before Present. These sites are located in North East Europe, a region that displays a significant cultural and linguistic diversity today but for which no ancient human DNA was available before. We show that prehistoric hunter-gatherers of North East Europe were genetically similar to other European foragers. We also detected a prehistoric genetic input from Siberia, followed by migrations from Western Europe into North East Europe. Our research contributes to the understanding of the origins and past dynamics of human population in Europe.

## Introduction

Our current knowledge of the origins of human populations and their migratory history relies on archaeological, anthropological, linguistic and genetic research. The study of genetic markers, especially the maternally inherited mitochondrial DNA (mtDNA), has allowed important events in the genetic history of humans to be reconstructed [1]–[11]. However, reconstructions based solely on present-day genetic diversity can be biased by a variety of evolutionary mechanisms, such as genetic drift and/or past population events. The ability to accurately reconstruct recent human evolutionary events can be significantly improved through the direct analysis of ancient human remains from representative time periods.

The mtDNA diversity of prehistoric populations has been previously described for Palaeolithic/Mesolithic hunter-gatherers from Central, Eastern and Scandinavian Europe [12]–[14], and for Neolithic farmers from Southern and Central Europe (CE) [15]–[20]. These studies have uncovered an unexpected and substantial heterogeneity in the geographical, temporal and cultural distribution of the mtDNA diversity. However, little is known about past mtDNA diversity in North East Europe (NEE), including the Baltic region, the Volga-Ural Basin (VUB), and sub-Arctic Europe. It is likely that different demographic events have been involved in shaping the gene pools of the populations of Western/Central Europe and NEE, due to the geographical position and distinct climatic conditions of the latter.

During the Upper Palaeolithic (∼30,000–40,000 years before present, yBP), the northernmost latitudes of Europe were covered by an ice sheet that prevented settlement by anatomically modern humans. With the glacial retreat at the end of the Ice Age (∼11,500 yBP) [21], small foraging groups progressed into NEE from southern periglacial refuges [22]–[23]. As climatic conditions improved in the early Holocene (8,000–10,000 yBP), the first human settlements appeared in the Kola Peninsula [24], and foraging activities intensified in the steppe-forest zone of Northern Europe leading to the widespread establishment of complex Mesolithic societies of fishermen and hunter-gatherers [23], [25]–[26]. At the same time, Western Europe and CE were undergoing the Neolithic transition, during which an agricultural lifestyle spread rapidly, largely due to favorable climatic and ecological conditions. The Neolithic transition is thought to have been slower and more gradual in NEE than in Western/Central Europe and to have involved little migration of early farmers from CE [27]. From the Neolithic onwards, contacts between populations of NEE and groups living in the South are evident in archaeological and historical records [24]. Around the Baltic, historical records describe numerous population movements that originated in Scandinavia (e.g., Viking incursions ∼800 Anno Domini, AD [28]), Western/Central Europe (e.g., the Slavic migrations ∼700–1,000 AD [29]) or Central/East Siberia (e.g., the Mongol invasions ∼500–700 AD [30]).

The geographical position of NEE makes it subject to influences from both Western and Eastern Eurasia, which could explain the linguistic and cultural diversity, observed in the area today. Two different linguistic families are spoken: Indo-European languages (Slavic, Baltic and Germanic) and Finno-Ugric languages (e.g., Estonian, Finnish, Mari, Saami [31]). Saami people of Fennoscandia (northern Norway, Sweden, Finland and Russia) are considered unique among Europeans in terms of their nomadic lifestyle and their livelihood, which is mainly based on fishing and reindeer herding. The ethnogenesis of the Saami remains unclear and two origins in Western and Eastern Europe were proposed [24], [32]–[33]. The Saami differ from the rest of the European populations in their reduced genetic diversity [1], [34]–[35], and mtDNA lineages that are otherwise very rare in European populations (haplogroups or hgs, U5b1b1a, V, Z1 and D5). In particular, the Saami-specific U5b1b1a clade is defined by the so-called hypervariable region I (HVR-I) ‘Saami motif’ 16144C-16189C-16270T (numbering according [36]) [37]. These lineages are also detected at low frequencies in adjacent NEE populations [32], [38]–[40], which on the other hand fall within the European mtDNA diversity and appear rather homogeneous irrespective of their languages [3]–[5], [38], [40]. Subtle mtDNA differences are however observed among them due to variable influences from genetically differentiated neighboring populations: central Europeans in the West, Saami in the North, and people from the VUB in the East.

**Figure 1:**
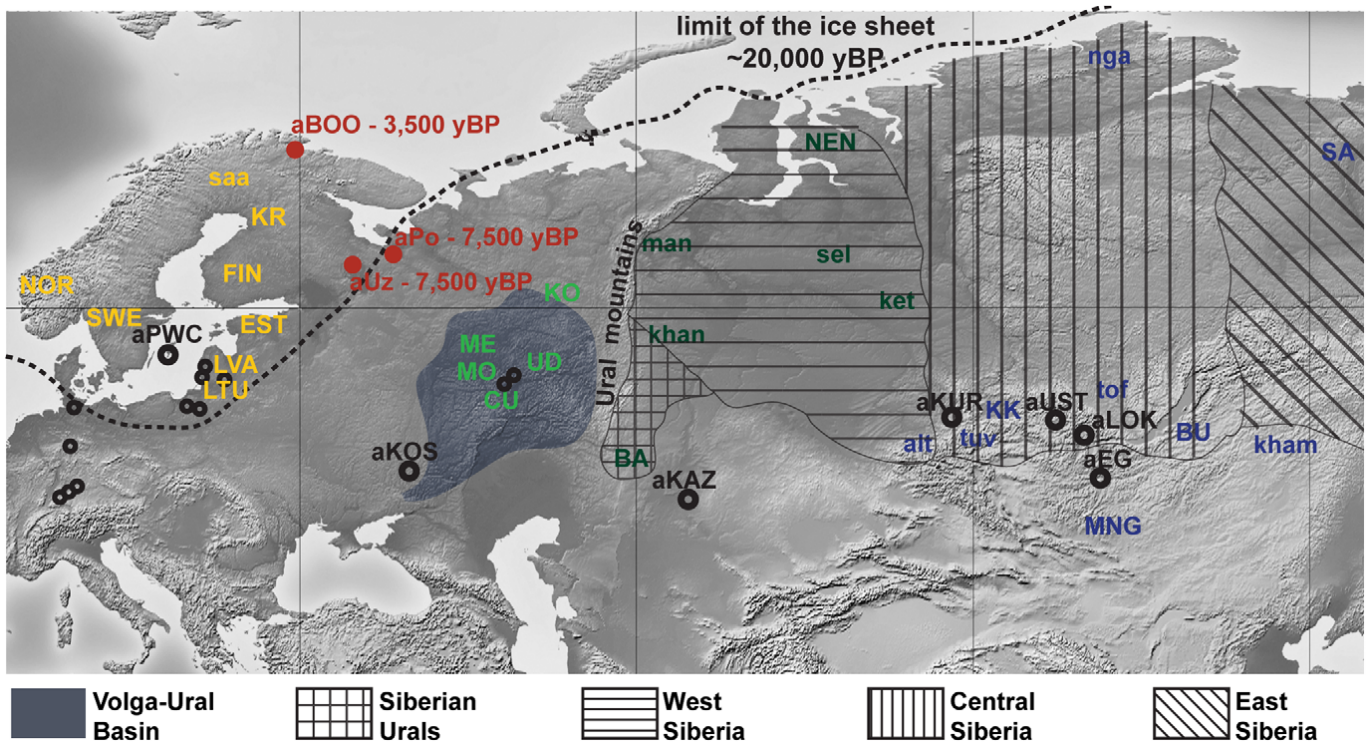
Map of Eurasia showing the approximate location of ancient (uncalibrated dates) and present-day Eurasian samples. Red dots represent the archaeological sites sampled for ancient mitochondrial DNA in this study: aUZ, Yuzhnyy Oleni Ostrov; aPo, Popovo; aBOO, Bol'shoy Oleni Ostrov. Black circles represent ancient populations abbreviated as follows: aEG, Confederated nomads of the Xiongnu (2,200–2,300 yBP); aKAZ, Nomads from Kazakhstan (2,100–3,400 yBP); aKOS, Kostenski individual (30,000 yBP); aKUR, Siberian Kurgans (1,600–3,800 yBP); aLOK, Lokomotiv Kitoi Neolithic individuals (6,130–7,140 yBP); aPWC, Scandinavian Pitted-Ware Culture foragers (4,500–5,300 yBP); aUST, Ust'Ida Neolithic population (4,000–5,800 yBP). Smaller black dots signify the location of Palaeolithic/Mesolithic sites sampled for ancient mitochondrial DNA in aHG (4,250–15,400 yBP). Present-day populations are abbreviated as follows: alt, Altaians; BA, Bashkirs; BU, Buryats; CU, Chuvash; EST, Estonians; FIN, Finns; ket, Kets; kham, Khamnigans; khan, Khants; KK, Khakhassians; KO, Komis; KR, Karelians; LTU, Lithuanians; LVA, Latvians; man, Mansi; ME, Mari; MO, Mordvinians; MNG, Mongolians; NEN, Nenets; nga, Nganasans; NOR, Norwegians; tof, Tofalars; tuv, Tuvinians; UD, Udmurts; SA, Yakuts; saa, Saami; sel, Selkups; SWE, Swedes. The approximate location of the Volga-Ural Basin and of the different regions of Russian Siberia are also indicated.

The absence of strong structure in the present-day mtDNA gene pool of NEE stands in contrast to the variety of languages and cultures, and to the complex history of how and when these were formed. Modern mtDNA data does not resolve the origins of the Saami either. Our aim was to provide answers to these questions and reconstruct events in the genetic history of NEE by generating and analyzing ancient DNA (aDNA) data from prehistoric human remains collected in northwest Russia (Figure 1). In particular, our objective was to characterize the genetic relationships between hunter-gatherer populations in NEE and Central/Northern Europe and to estimate the genetic legacy of ancient populations to present-day NEE and Saami. The oldest samples were collected in the Mesolithic graveyards of Yuzhnyy Oleni Ostrov (aUz; ‘Southern Reindeer Island’ in Russian) and Popovo (aPo), both dated around 7,000–7,500 uncalibrated. yBP, uncal. yBP. The sites of aUz and aPo are located along one of the proposed eastern routes for the introduction of Saami-specific mtDNA lineages [32]. Results from odontometric analyses suggested a direct genetic continuity between the Mesolithic population of Yuzhnyy Oleni Ostrov and present-day Saami [41]. We also analyzed human remains from 3,500 uncal. yBP site Bol'shoy Oleni Ostrov (aBOO; ‘Great Reindeer island’ in Russian) in the Kola Peninsula. This site is located within the area currently inhabited by the Saami. We compared the ancient mtDNA data from NEE with a large dataset of ancient and modern-day Eurasian populations to search for evidence of past demographic events and temporal patterns of genetic continuity and discontinuity in Europe.

## Results

### Amplification success and authentication of the ancient DNA data

The skeletal remains from aUz, aPo, and aBOO were genetically analysed by i) direct sequencing of the mtDNA hyper-variable region I (HVR-I, nucleotide positions, np 16056–16409) and ii) typing of 22 haplogroup-diagnostic single nucleotide polymorphisms (SNPs) in the coding-region using the GenoCore22 reaction [16]. Strict criteria were followed to authenticate aDNA data and detect contamination by exogenous DNA or artefactual mutations caused by post-mortem DNA damage (see Materials and Methods). In total, 34 ancient genotypes were obtained that were considered unambiguous on the basis of these authenticity criteria (Table 1). Sequences have been deposited in Genbank (http://www.ncbi.nlm.nih.gov/genbank/; accession numbers KC414891-KC414924).

**Table 1:** Results for mitochondrial DNA typing.

Sample	HVR-I sequence^a^ (np 16056–16409), Minus np 16000	Hg (HVR-I)	Hg (GenoCore22)	Analyses^b^
**7,500 uncal. yBP Yuzhnyy Oleni Ostrov (61°30′N 35°45′E)**				
UZOO-43	129c-189C-362C	U2e	U	E(2), Q
UZOO-46	129c-189C-362C	U2e	U	E(2)
UZOO-16	093C-356C	U4	U	E(2)
UZOO-40	093C-356C	U4	U	E(2)
UZOO-70	192T-256T-270T-318G	U5a	U	E(2)
UZOO-77	235G-311C-362C	H	H	E(2), I, C(22)
UZOO-7	189C-223T-298C-325C-327T	C1	C	E(2)
UZOO-8	189C-223T-298C-325C-327T	C1	C	E(2)
UZOO-74	189C-223T-298C-325C-327T	C1	C	E(2), Q
**7,000 uncal. yBP Popovo (64°32′N 40°32′E)**				
Po4	356C	U4	U	E(2)
Po2	093C-356C	U4	U	E(2)
**3,500 uncal. yBP Bol'shoy (68°58′N 35°05′E)**				
BOO49-3	093C-129A-134T-311C-356C	U4a1	U	E(2)
BOO57-1	093C-129A-134T-311C-356C-(390R)^c^	U4a1	U	E(2), I, C(8)
BOO49-1	192T-256T-270T	U5a	U	E(2)
BOO72-11	192T-256T-270T	U5a	U	E(2)
BOO72-9	192T-256T-270T-399G	U5a1	U	E(1), Q
BOO72-10	192T-256T-270T-399G	U5a1	U	E(2)
BOO72-14	192T-256T-270T-399G	U5a1	U	E(2)
BOO72-8	192T-256T-270T-399G	U5a1	U	E(2)
BOO72-4	093C-126C-294T	T*	T	E(2), I, C(6)
BOO49-2	223T-298C-327T	C*	C	E(2)
BOO49-4	223T-298C-327T	C*	C	E(2)
BOO57-3	223T-298C-327T	C*	C	E(2)
BOO72-2	223T-298C-327T	C*	C	E(2)
BOO72-7	223T-298C-327T	C*	C	E(2) I, C(4)
BOO72-12	223T-298C-327T	C*	C	E(2)
BOO72-5	148T-223T-288C-298C-311C-327T	C5	C	E(2)
BOO72-6	148T-223T-288C-298C-311C-327T	C5	C	E(2)
BOO49-6	223T-362C	D*	D	E(2)
BOO72-13	223T-362C	D*	D	E(2)
BOO72-15	223T-362C	D*	D	E(2) I, C(5)
BOO49-5	129A-185T-223T-224C-260T-298C	Z1a	M	E(2)
BOO72-3	129A-185T-223T-224C-260T-298C	Z1a	M	E(2)
BOO72-1	129A-155G-185T-223T-224C-260T-298C	Z1a	M	E(2), I, C(6), Q

a Transitions are reported with upper-case letters, transversions with lower-case letters.

b E, number of samples from which DNA was independently extracted; I, results replicated in an independent laboratory; C, number of HVR-I clones; Q, HVR-I DNA quantification performed.

c Position 390R was not included in population genetics analyses.

Hg, haplogroup; HVR-I, hypervariable region I; np, nucleotide positions; yBP, years Before Present.

The success of DNA amplification reactions varied among archaeological sites as follows: 9/42 individuals (21.5%) for aUz, 2/3 (66.7%) for aPo, and 23/23 (100.0%) for aBOO. The higher success rates (100%) observed for samples from aBOO were consistent with their younger age and excellent macroscopic preservation, probably due to the cold climatic conditions of the Kola Peninsula (Figure S1). The presence of naturally crushed marine shells in the burial grounds of aBOO has also been proposed to explain the exceptional preservation of the remains [42]. In contrast, and in accordance with their poorer macroscopic preservation, aDNA from the samples of aUz and aPo was more difficult to amplify, with a lower amplification success and some contaminated results that had to be excluded.

### Haplogroup distribution in modern-day populations of Eurasia

**Figure 2:**
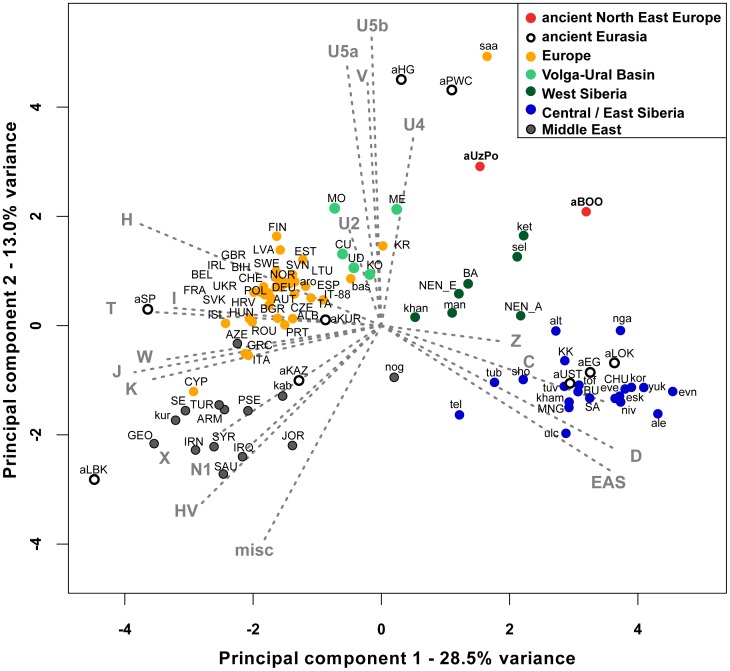
Principal Component Analysis of mitochondrial haplogroup frequencies. The first two dimensions account for 41.5% of the total variance. Grey arrows represent haplogroup loading vectors, i.e., the contribution of each haplogroup. Red dots represent ancient populations described in this study: aUzPo, Yuzhnyy Oleni Ostrov and Popovo (7,500 uncal. yBP); aBOO, Bol'shoy Oleni Ostrov (3,500 uncal. yBP). Other ancient populations were labeled as follows: aEG, Confederated nomads of the Xiongnu (4,250-2,300 yBP); aHG, Palaeolithic/Mesolithic hunter-gatherers of Central/East Europe (4,250-30,000 yBP); aKAZ, Nomads from Kazakhstan (2,100–3,400 yBP); aKUR, Siberian Kurgans (1,600–3,800 yBP); aLBK, Neolithic individuals from Germany (7,000–7,500 yBP); aLOK, Lokomotiv Kitoi Neolithic individuals (6,130–7,140 yBP); aSP, Neolithic individuals from Spain (5,000–5,500 yBP); aPWC, Scandinavian Pitted-Ware Culture foragers (4,500–5,300 yBP); aUST, Ust'Ida Neolithic population (4,000–5,800 yBP). Extant populations were abbreviated as follows: ALB, Albanians; ale, Aleuts; alt, Altaians; ARM, Armenians; aro, Arorums; AUT, Austrians; AZE, Azerbaijani; BA, Bashkirs; bas, Basques; BEL, Belarusians; BGR, Bulgarians; BIH, Bosnians; BU, Buryats; CHE, Swiss; CHU, Chukchi; CU, Chuvashes; CYP, Cypriots; CZE, Czechs; DEU, Germans; esk, Eskimos; ESP, Spanish; EST, Estonians; eve, Evenks; evn, Evens; FIN, Finns; FRA, French; GBR, British; GEO, Georgians; GRC, Greeks; HRV, Croatians; HUN, Hungarians; ing, Ingrians; IRL, Irish; IRN, Iranians; IRQ, Iraqi; ISL, Icelanders; IT-88, Sardinians; ITA, Italians; JOR, Jordanians; kab, Kabardians; ket, Kets; kham, Khamnigans; khan, Khants; KK, Khakhassians; KO, Komi; kor, Koryaks; KR, Karelians; kur, Kurds; LTU, Lithuanians; LVA, Latvians; man, Mansi; ME, Mari; MNG, Mongolians; MO, Mordvinians; NEN_A, eastern Nenets; NEN_E, western Nenets; nga, Nganasans; niv, Nivkhs; nog, Nogays; NOR, Norwegians; POL, Poles; PRT, Portuguese; PSE, Palestinans; ROU, Romanians; RUS, Russians; SA, Yakuts; saa, Saami; SAU, Saudi Arabians; SE, Ossets; sel, Selkups; sho, Shors; SVK, Slovakians; SVN, Slovenians; SWE, Swedes; SYR, Syrians; TA, Tatars; tel, Telenghits; tof, Tofalars; tub, Tubalars; TUR, Turks; tuv, Tuvinians; UD, Udmurts; UKR, Ukrainians; ulc, Ulchi; vep, Vepses; yuk, Yukaghirs.

In order to identify the genetic affinities of the two ancient populations with other ancient and present-day Eurasian populations, mtDNA hg distributions were compared by Principal Component Analysis (PCA). The PCA plot of the first two components (41.5% of the total variance, Figure 2) showed that present-day populations largely segregate into three main clusters: Europeans (in yellow), Middle Easterners (in grey) and Central/East Siberians (in blue). The spread of extant populations of Europe and Central/East Siberia along the first component axis (28.5% of the variance) appeared to reflect their longitudinal position, whereas Europeans and Middle Easterners were separated along the second component axis (13.0% of the variance). As shown previously, populations of the ‘Central/East Siberian’ cluster were predominantly composed of hgs A, B, C, D, F, G, Y, and Z, while in contrast populations of the ‘European’ cluster were characterized by higher frequencies of hgs H, HV, V, U, K, J, T, W, X, and I (e.g., [43]–[47]). The two ancient groups - aUzPo and aBOO - from two individual time periods appeared remarkably distinct on the basis of the PCA, suggesting a major genetic discontinuity in space and time.

### Comparison of Mesolithic Yuzhnyy Oleni Ostrov/Popovo (aUzPo) with extant populations of Eurasia

**Figure 3:**
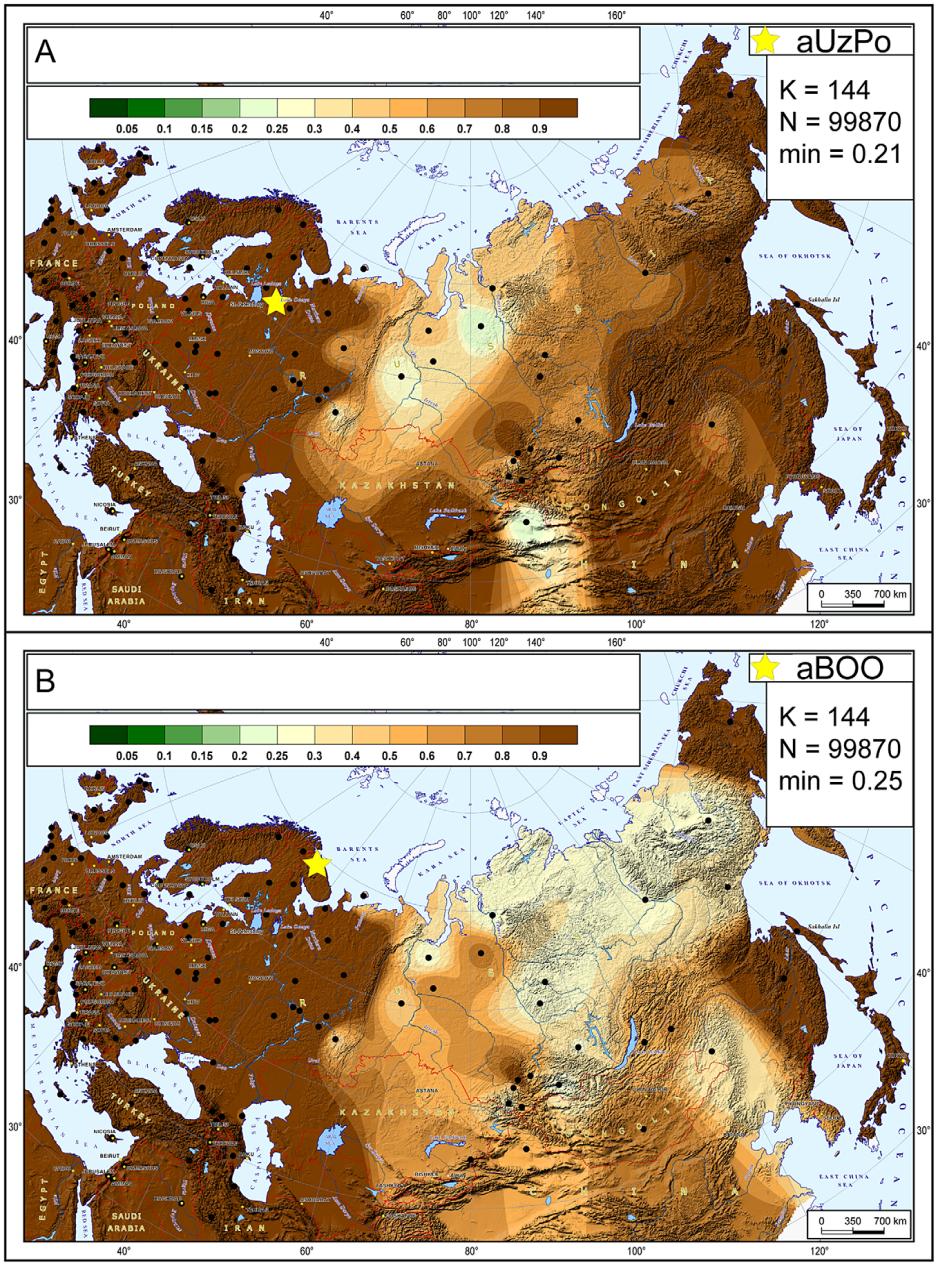
Map of genetic distances between modern-day populations of Eurasia and from aUzPo and aBOO. Genetic distances were computed between 144 modern-day populations geographically delineated across Eurasia (red dots) and the eleven individuals from aUzPo (A) and the 23 individuals from aBOO (B). The colour gradient represents the degree of similarity between the modern and ancient populations, interpolated between sampling points: from ‘green’ for high similarity or small genetic distance to ‘brown’ for low similarity. ‘K’ designates the number of populations used for distance computation and mapping; ‘N’ represents the number of points in the grid used for extrapolation; ‘min’, corresponds to the minimal values respectively of the computed distances between ancient and modern populations.

The hg distribution in the Mesolithic aUzPo population: U4 (37%), C (27%), U2e (18%), U5a (9%), and H (9%), indicated an ‘admixed’ composition of ‘European’ (U4, U2e, U5a and H, 73%) and ‘Central/East Siberian’ (C, 27%) hgs, based on the PCA plot (Figure 2). Interestingly, the population of aUzPo did not group with modern NEE populations, including Saami, but fell instead between the present-day ‘European’ and ‘Central/East Siberian’ clusters on the PCA graph, and more precisely between populations of the VUB (in light green) and West Siberia (in dark green). The high frequency of hg U4 is a feature shared between Mesolithic aUzPo, present-day VUB (Komi, Chuvashes, Mari), and West Siberian populations (Kets, Selkups, Mansi, Khants, Nenets), with the latter group also being characterized, like aUzPo, by the presence of hg C. The genetic affinity between Mesolithic aUzPo and present-day West Siberian populations could be visualized on the genetic distance map of North Eurasia (Figure 3A), on which locally lighter colorings indicated low values of genetic distances, and therefore an affinity between aUzPo and extant West Siberians.

**Figure 4:**
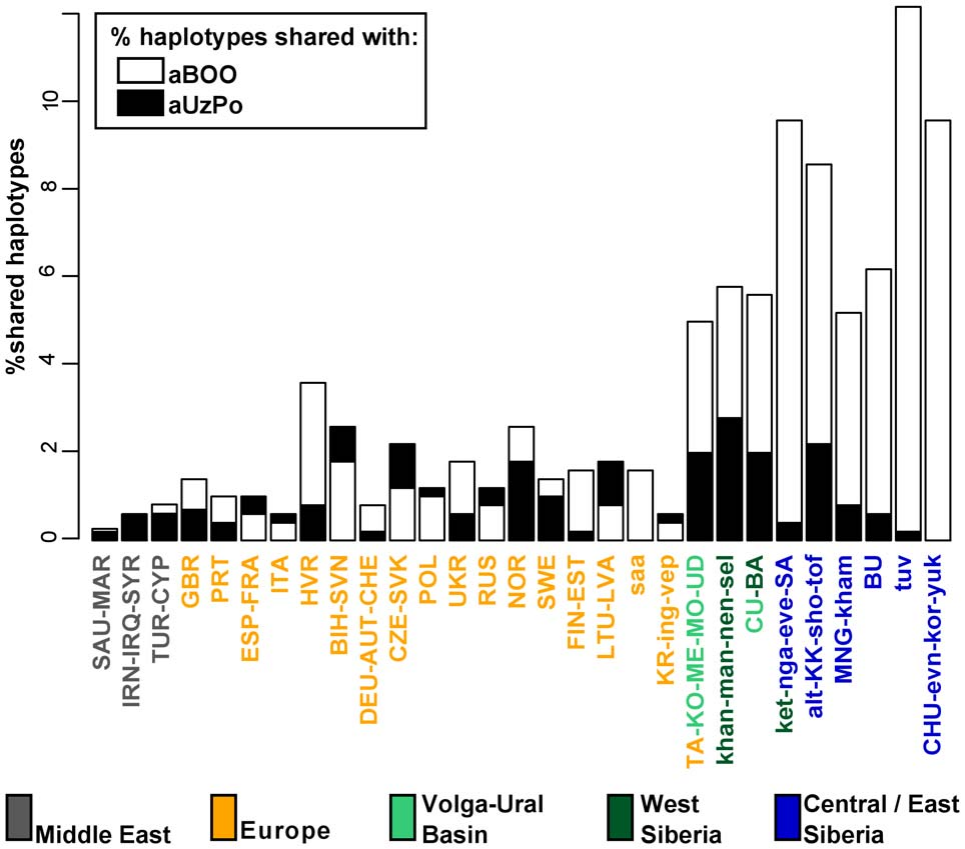
Percentages of haplotypes from aUzPo and aBOO matched in modern-day Eurasian population pools. Percentages of matches for the haplotypes from aBOO are represented by white bars. Percentages of matches for the haplotypes from aUzPo are independently represented by superimposed black bars.

In order to test the potential population affinities formulated on the basis of the hg-frequency PCA and the distance map, we examined the present-day geographical distribution of the haplotypes found in aUzPo via haplotype sharing analyses (Figure 4). These analyses are less impacted by biases due to small population sizes or unidentified maternal relationships in ancient populations, and thus are less prone to artefacts. Although the highest percentages of shared haplotypes for aUzPo were observed in pools of West Siberian Khants/Mansi/Nenets/Selkups (2.8%), South Siberian Altaians/Khakhassians/Shors/Tofalars (2.2%) and Urals populations (Chuvash/Bashkirs, 2.0%), matches were widely distributed across Eurasia. This was consistent with the observation that most haplotypes sequenced in aUzPo were basal and hence, not informative in terms of geographical population affinity. Haplogroup-based analyses suggested that the genetic affinity between aUzPo and present-day West Siberians was partly due to the presence of hg C, implying that the non-basal haplotype C1 found in aUzPo (16189C-16223T-16298C-16325C-16327T, detected in three individuals) could be a clear genetic link with extant Siberian populations. However, the C1 haplotype found in aUz did not belong to hg C1a, the only C1 clade restricted to Asia (characterized by a transition at np 16356 [48]). Indeed, no exact match was found for the C1 haplotype in the comparative database of Eurasian populations (comprising 168,000 haplotypes), although 47 derivatives (showing one to three np differences) were found in extant populations broadly distributed throughout Eurasia (Table S1). Therefore, the C1 haplotype sequenced in aUzPo is currently uninformative about population affinity. In addition, all three aUzPo individuals showed identical C1 haplotypes, which meant that a close maternal kinship between these individuals could not be rejected. Biases due to the overestimation of the hg C1 frequency and small sample size of aUzPo may have led to an overestimation of the genetic affinity with modern-day West Siberians in the hg-based analyses. To account for this, we assumed a scenario of extreme maternal kinship, in which identical haplotypes found in several individuals at the same site (redundant haplotypes) were only counted once (Figure S2A). Under this scenario, the genetic affinity between aUzPo and present-day Western Siberians was less distinctly pronounced (Figure S2B).

**Figure 5:**
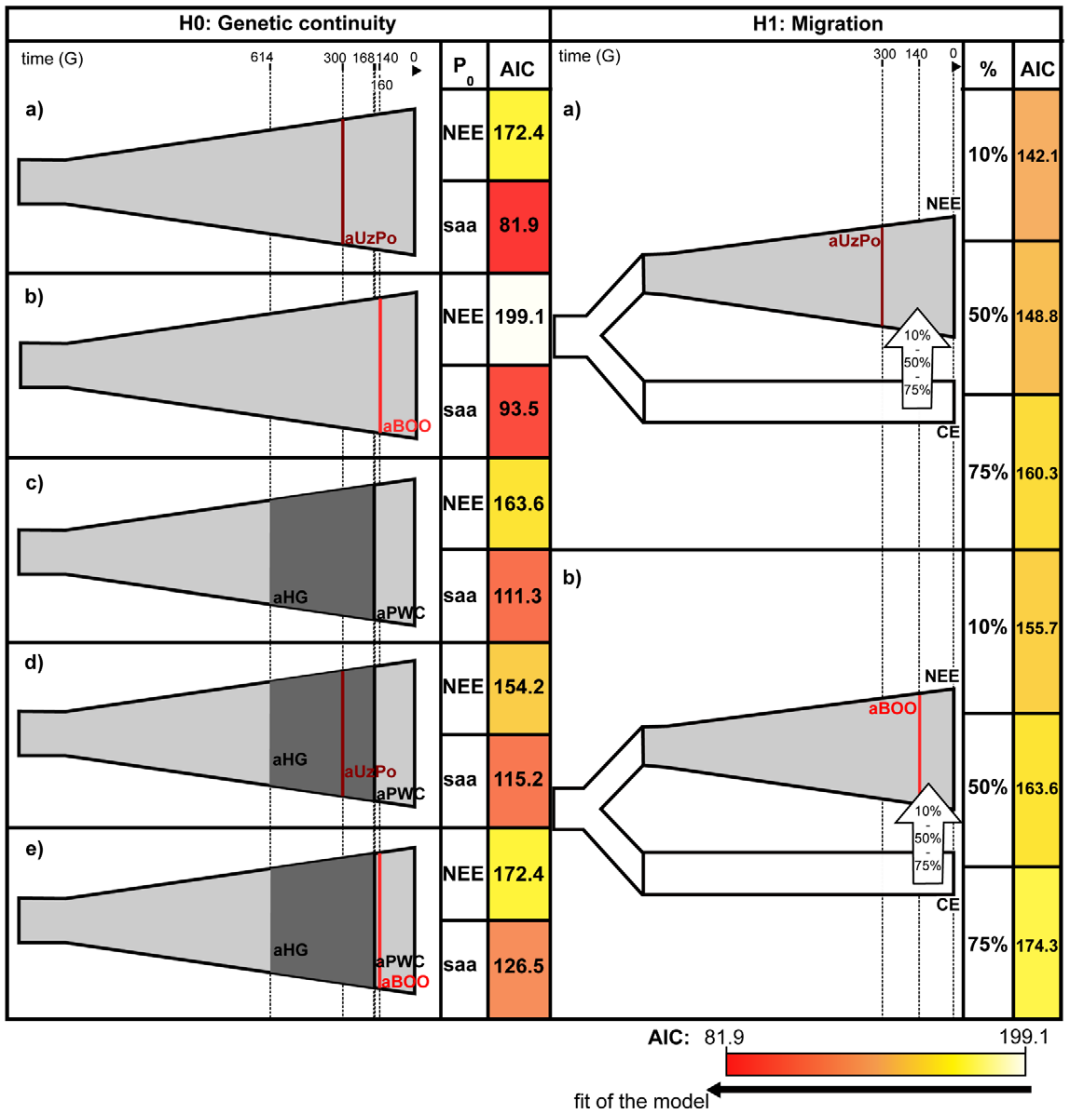
Graphical representation and Akaike Information Criterions of the demographic models compared by coalescent simulation analyses. The timeline indicates the age of populations in generations (G). For models H0a to H0e, genetic continuity is tested between combinations of ancient populations and present-day populations of North East Europe (NEE) or Saami (saa), as indicated in the column ‘P_0_’. For models H1a and H1b, genetic discontinuity between aUzPo or aBOO, and NEE is tested assuming a migration from Central Europe (CE). The percentage of migrants from the source population into the sink population (10%, 50% and 75%) is indicated in the column ‘%’. The cells containing Akaike Information Criterion (AIC) values were colored according to the gradient of AIC represented below the figure: from white for the highest value of AIC (worst model fit, 199.1 for H0b) to red for the lowest value of AIC (best model fit, 81.9 for H0a).

To further evaluate the apparent significant genetic discontinuity between aUzPo and modern extant populations of NEE and Saami, we analyzed Bayesian Serial SimCoal (BayeSSC) coalescent simulations [49] using Approximate Bayesian Computation (ABC, [50]) and tested whether discontinuity could be better explained by genetic drift or by migration. Models of genetic continuity between aUzPo and the present-day population of NEE or Saami (H0a) were compared to models in which genetic discontinuity between aUzPo and the extant population of NEE was introduced by migration (H1a, Figure 5). Ancestors of individuals from CE were selected as a source population for the migration on the basis of the PCA plot (Figure 2) showing that present-day populations of NEE shared the most genetic similarities with those of CE. The model of genetic discontinuity between aUzPo and present-day Saami was not tested since no source population for a potential migration could be identified from the PCA plot. The model of genetic continuity between aUzPo and present-day Saami was found to fit the observed data better than the model of genetic continuity between aUzPo and present-day NEE. This can be attributed to the low haplotypic diversities (0.74 and 0.81, respectively, in contrast to 0.98 for NEE; Table 2) of both aUzPo and Saami populations. The migration model provided a better fit for the genetic data than the model of genetic continuity (H0a), as indicated by a low Akaike Information Criterion (AIC, [51]) and a high Akaike weight ω [52]–[53]. The lowest AIC (Figure 5) and highest Akaike's ω (Table 3) were obtained for migration models, the best fit being obtained for the model involving 10% of migrants over the last 7,500 years (H1b; ω = 1.00E+0 as opposed to ω = 2.57E-7 for the continuity model H0a). Our analyses of coalescent simulations therefore supported a genetic discontinuity between aUzPo and the present-day population of NEE, which was better explained by a migration from CE than by genetic drift.

**Table 2:** Population parameters and statistics used in Bayesian Serial SimCoal coalescent simulation analyses.

	North East Europeans	Saami	Central Europeans	European hunter-gatherers	YuzhnyyOleni Ostrov	Bol'shoy Oleni Ostrov	Pitted Ware Culture hunter-gatherers
**Abbreviation**	NEE	saa	CE	aHG	aUzPo	aBOO	aPWC
**Sample size**	621	118	1030	20	11	23	19
**Age (generations)**	0	0	0	168 – 614	300	140	116
**Haplotype diversity**	0.98	0.81	0.97	0.91	0.74	0.82	0.80
**Fixation indices F_ST_**							
**NEE**	-						
**saa**	0.1245	-					
**CE**	0.0040	n/a	-				
**aHG**	0.0765	0.1879	n/a	-			
**aUzPo**	0.0538	0.1391	0.6564	0.1520	-		
**aBOO**	0.1232	0.1698	0.1418	0.1680	0.0577	-	
**aPWC**	0.0507	0.1993	n/a	0.0943	0.0630	0.1723	-

**Table 3:** Relative model likelihood of the demographic models simulated in Bayesian Serial SimCoal, as indicated by Akaike weights ω.

Genetic continuity with NEE (H0) versus migration from CE (H1)		Akaike weights ω
**aUzPo**	a) H0	2.57 E-7
	a) H1 with 10% migrants	1.00 E+0
**aBOO**	b) H0	3.86 E-10
	b) H1(10% migrants)	1.00 E+0

Genetic continuity (H0)		Akaike weights ω
**with NEE**	b) H0: aBOO	1.76 E-10
	a) H0: aUzPo	1.06 E-4
	e) H0: aBOO+aHG+aPWC	1.10 E-4
	c) H0: aHG+aPWC	9.05 E-3
	d) H0: aUzPo+aHG+aPWC	9.91 E-1
**with saa**	e) H0: aBOO+aHG+aPWC	2.04 E-10
	d) H0: aUzPo+aHG+aPWC	5.86 E-8
	c) H0: aHG+aPWC	4.23 E-7
	b) H0: aBOO	3.09 E-3
	a) H0: aUzPo	9.97 E-1

Percentages of migrants (H1)		Akaike weights ω
**aUzPo**	a) H0: 75%	1.06 E-4
	a) H0: 50%	3.33 E-2
	a) H0: 10%	9.67 E-1
**aBOO**	b) H0: 75%	9.20 E-4
	b) H0: 50%	1.92 E-2
	b) H0: 10%	9.81 E-1

For each hypothesis tested, models are ordered from the least likely (lowest ω) to the most likely model (highest ω). NEE, North East Europe; CE, Central Europe.

### Comparison of 3,500 uncal. yBP Bol'shoy Oleni Ostrov (aBOO) with extant populations of Eurasia

At the 3,500 uncal. yBP site of aBOO, we observed 39% ‘European’ hgs: U5a (26%), U4 (9%), T (4%), and 61% ‘Central/East Siberian’ hgs: C (35%), Z (13%), D (13%). Concordant with this admixed hg make-up, PCA indicated a position close to present-day Siberians (Figure 2). This position did not change when potential maternal relationships among individuals were accounted for by excluding redundant haplotypes (Figure S2B). The genetic relationship between aBOO and Siberians was also evident on the genetic distance map, where the area representing the lowest genetic distance covered a broader area of Siberia than for aUzPo (Figure 3B). The extant populations that showed most genetic similarity to aBOO were found in Central and East Siberia. In contrast, the area of maximum similarity for aUzPo lay in West Siberia (Figure 3A); this observation however could be influenced by low sample size in aUzPo.

Haplotype sharing analyses for aBOO confirmed the genetic affinity with modern-day West and Central/East Siberians inferred from the PCA (Figure 4), but also identified a close relationship with the VUB population pool. The distribution of haplotype matches observed in pools of the VUB, West Siberia and Central/East Siberia was partly due to the presence of basal C* (16223T-16298C-16327T) and D* (16223T-16362C) haplotypes in these pools, whereas these types were absent in Middle Eastern and European pools. Central Siberian Tuvinians displayed the highest percentage of shared haplotypes with aBOO (12.2%) although all shared haplotypes belong to hgs C* and D*. A more explicit genetic link between aBOO and extant East Siberians was seen in the presence of the derived C5 haplotype (16148T-16223T-16288C-16298C-16311C-16327T) in aBOO and in one single Buryat individual of Central Siberia [54]. The Z1a haplotype (16129A-16185T-16223T-16224C-16260T-16298C) detected in aBOO had a broad but interesting distribution in Eurasia. It was found in all Central/East Siberian pools except in Tuvinians, but also in the Bashkirs of the Urals, in the VUB pool, as well as in Scandinavian and Baltic populations (Norwegians, Swedes, Finns, Ingrians, Karelians, and the Saami).

Although haplotype sharing analyses revealed genetic links between aBOO and extant populations of NEE, a strong genetic differentiation was obvious between aBOO, modern-day NEE and Saami. This genetic discontinuity was further supported by BayeSSC analyses (Figure 5; Table 3). Similarly to aUzPo, a better fit was obtained for the model involving a 10% migration from CE over the last 3,500 years (H1b; ω = 1.00E+0) than for the model of genetic continuity between aBOO and NEE (H0b; ω = 3.86E-10).

### Comparison among ancient Eurasian populations

Previously described populations of hunter-gatherers of Central/East Europe (aHG [12], [14]) and Scandinavia (aPWC, [13]) were characterized by high frequencies and diversity of hg U4, U5a and U5b, which caused the two ancient datasets to group outside the cluster of extant European populations on the PCA plot (Figure 2). This matches previous studies that have shown that genetic continuity between hunter-gatherers and present-day Europeans can be rejected [12]–[13]. Like other European hunter-gatherers, aUzPo is characterized by high frequencies and diversity of hgs U4 and U5, but was genetically differentiated from aHG and aPWC due to the occurrence of hg C. Despite the fact that high frequencies of hgs U5b and V cluster the aHG and aPWC hunter-gatherer groups on the PCA plot (Figure 2), and that these hgs are also common in modern-day Saami, the ‘Saami motif’ is absent from aPWC and genetic continuity between aPWC and modern-day Saami was rejected [13].

**Figure 6:**
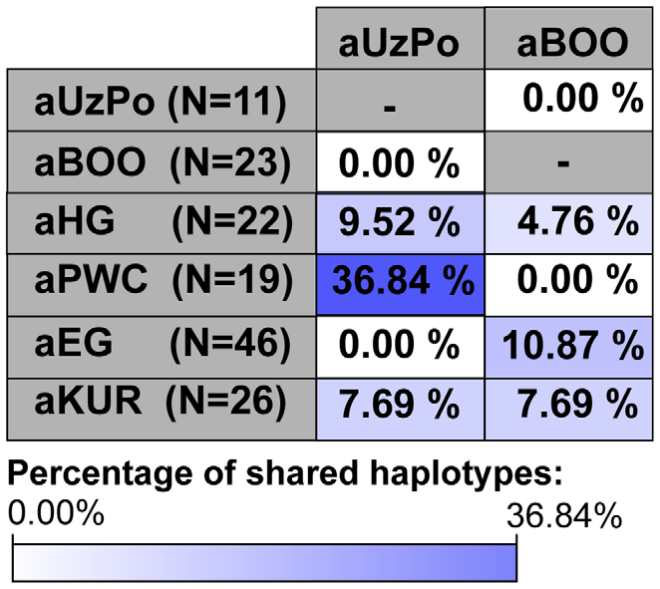
Percentages of haplotypes from aUzPo and aBOO matched in selected ancient Eurasian populations. The cells were colored according to the gradient of percentages of shared haplotypes represented below the figure: from white for the lowest value of percentages of shared haplotypes (0.00%) to dark blue for the highest value of percentages of shared haplotypes (36.84% between aUzPo and aPWC).

Although the aBOO individuals were also characterized by high frequencies of hg U, the group appeared less close to the Palaeolithic/Mesolithic hunter-gatherers aHG and aPWC on the PCA plot than aUzPo. Haplotype sharing analyses (Figure 6) also showed that aBOO shared less haplotypes with aHG and aPWC than aUzPo (4.76% and 0.00%, respectively, versus 9.52% and 36.84%). This observation was confirmed by the analyses of our coalescent simulations, in which a model of genetic continuity between aHG, aPWC and aUzPo (ω = 9.91 E-1; H0d) was better supported than a model of genetic continuity between aHG, aPWC and aBOO (ω = 1.10 E-4; H0e). As demonstrated above, aBOO exhibited greater genetic affinities with extant populations of Siberia than aUzPo. Accordingly, aBOO shared more haplotypes with ancient samples from Siberia aEG (10.87% [55]) and aKUR (7.69% [56]) than aUzPo (0.00% and 7.69%, respectively; Figure 6).

## Discussion

To date, all studies on ancient Mesolithic/Palaeolithic hunter-gatherers from Europe have reported large proportions of hg U: 64% in aUzPo, 73% in aHG, 74% in aPWC; and hg U was also found in three out of five Mesolithic individuals of Spain [20], [57]. On the basis of the distribution of hg U5b, it was proposed that the Mesolithic population has remained genetically homogeneous over a wide geographical area and for a long period of time [57]. The new data from aUzPo suggests that hg U5a may be a representative of Central and North East Europe's Mesolithic mtDNA diversity, whereas elevated frequencies of hg U4 appear more characteristic of populations of the peri-Baltic area (aUzPo and aPWC). Haplogroup U also represents a significant genetic component of aBOO (35%), as well as Bronze Age Central Asians (14% in aKAZ; 2,700–3,400 yBP), and pre-Iron Age Siberians (54% in aKUR; Andronovo and Karasuk cultures; 2,800–3,800 yBP). Today, hg U is found in 7% of Europeans and displays a wide distribution in Europe, West Siberia, south west Asia, the Near East and North Africa [5]. Both the widespread distribution and high variability of hg U in extant and prehistoric populations are consistent with the description of hg U as one of the oldest hgs in Europe. On the basis of modern genetic data, hg U was proposed to have originated in the Near East and spread throughout Eurasia during the initial peopling by anatomically modern humans in the early Upper Palaeolithic (around 45,000 yBP, [5]). It is then plausible that hg U constituted the major part of the Palaeolithic/Mesolithic mtDNA substratum from Southern, Central and North East Europe to Central Siberia. It can also be suggested that the Palaeolithic/Mesolithic mtDNA substratum has been preserved longer in NEE than in Central and southern parts of Europe, where new lineages arrived with incoming farmers during the Neolithisation from the Near East [16]. This is supported by ancient genomic data obtained from hunter-gatherers of Scandinavia [58] and Spain [57], that shows a genetic affinity between Mesolithic individuals and present-day northern Europeans and supports genetic discontinuity between Mesolithic and Neolithic populations of Europe.

The detection of haplogroup H in the Mesolithic site of aUz (one haplotype) is noteworthy. To date, haplogroup H has either been rare or absent in groups of hunter-gatherers previously described. It has not been found in hunter-gatherer mtDNA datasets of eastern Europe [12] and Scandinavia [13], but has been found in two hunter-gatherers of the Upper Palaeolithic sites of La Pasiega and La Chora in northern Spain [20]. The closest match to the ancient H haplotype in aUzPo belongs to sub-haplogroup H2a2 [59], which is more common in eastern Europe [60] with highest frequencies in the Caucasus. Current ancient data is too scarce to investigate the past phylogeography of haplogroup H in full detail. However, together with U4, U5 haplotypes this H haplotype suggests continuity of some maternal lineages in (North) East Europe since the Mesolithic.

While the Mesolithic aUzPo site showed genetic affinities with extant populations of West Siberia in hg-based analyses, the precise genetic origins of aUzPo individuals was more difficult to identify from haplotypic data due to the high number of basal haplotypes. At the archaeological level also, the Siberian connection with aUzPo is less clear. The material culture present in the burials of aUz links these populations with the neighboring regions in the West but also in the East and South-East [26], [61]. As for Siberia, it has undergone a complicated early and mid-Holocene migration history due to repeated environmental changes [62]. With the data at hand, it is therefore difficult to make any definite statement about sixth millennium connections between Karelia and Siberia.

Interestingly, samples from aBOO, which are 4,000 years younger and located further North-West than aUzPo, were characterized by a large proportion and elevated diversity of mtDNA lineages showing a clear ‘Central/East Siberian’ origin (hgs C, D, and Z). Haplogroups C and D are the most common hgs in northern, central and eastern Asia. They are thought to have originated in eastern Asia and expanded through multiple migrations after the Late Glacial Maximum (∼20,000 yBP [63]). Notably, haplotypic matches were observed between aBOO and modern-day central Siberian Buryats of the peri-Baikal region, which was proposed to be the origin of ancient migrations that disseminated hgs C and D [63]. Today, the sharp western boundary for the distribution of hgs C, D and Z lies in the VUB, where they display intermediate frequencies: C (0.3–11.8%), Z (0.2–0.9%), and D (0.6–12%) [64]. Sub-hgs Z1 and D5 are also present in modern-day Saami, with highest cumulated frequencies (15.9%) in the Saami of Finland, the easternmost part of the Saami geographical distribution [32]. A precise date for the arrival of these ‘Central/East Siberian’ lineages in NEE is difficult to estimate, although the presence of ‘Central/East Siberian’ lineages in the 3,500 year-old aBOO site indicates that an eastern genetic influence pre-dates historical westward expansions from Central/East Siberia of, e.g., the Huns and the Mongols (∼400–1,500 AD). We present here direct genetic evidence for a prehistoric gene-flow from Siberia. On the basis of modern-day genetic data, hg Z1 was proposed to have been introduced into populations of the VUB and Saami by migrations from Siberia via the southern Urals to the Pechora and Vychegda basins (northwest Urals), associated with the appearance of the Kama culture ∼8,000 yBP [22], [32]. The presence of hg Z1 in aBOO establishes a direct genetic link between aBOO and modern-day populations of the VUB and Saami, and possibly indicates the trajectory of the migration that brought ‘Central/East Siberian’ lineages into NEE. The fact that aBOO did not contain any other Saami-specific haplotypes, suggests an independent origin and contribution of Z1 to the Saami gene pool.

The genetic links between the sample populations of aUzPo/aBOO and the extant populations of Siberia follow a general pattern discussed for the early and mid-Holocene (6,000–10,000 yBP). Facilitated by the East-West extension of vegetation zones between the Russian Far East and Eastern Europe [65], long-distance contacts and connections across Eurasia have been proposed for a number of cases. For example, the North East and East European hunter-gatherer pottery is thought to have originated in the early ceramic traditions of the Russian Far East and Siberia [66]–[68]. An eastern Asian origin followed by a westward expansion was also discussed for domesticated broomcorn millet (*Panicum miliaceum L.*) [69]. While the exact scenario behind these two examples of long-distance connections is unclear, migrations are a common interpretative model for evidence from later periods [70]. In any case, long-distance connections across Eurasia are not unusual. A later migration from the East was associated with the spread of the Imiyakhtakhskaya culture from Yakutia (East Siberia) through northwestern Siberia to the Kola Peninsula during the Early Metal Age (3,000–4,000 yBP, [24]). Interestingly, one individual of the aBOO site (grave 10, not sampled for aDNA here) was archaeologically associated with this culture, but its cultural relationships to other individuals of the same site remain unclear.

The apparent genetic discontinuity between aUzPo and aBOO is consistent with craniometrical analysis that have proposed a genetic discontinuity between the two groups despite the finding of ‘caucasoid’ and unusual ‘mongoloid’ cranial features at both sites [28]. Samples of aBOO were also shown to display craniometrical affinities with ancient populations of West Siberia and the Altai, in line with the ancient genetic data presented here [42]. The ‘admixed’ nature of the aUzPo and aBOO populations is supported by the apparent random distribution of mtDNA lineages within the corresponding graveyards, i.e., there is no structure in the sites reflecting the ‘Western’ or ‘Eastern’ origins of the buried individuals [26].

The present-day Saami populations display clear haplotypic differences from all the ancient populations sampled for DNA so far (prehistoric hunter-gatherer populations of North/South/Central/East Europe, aUzPo and aBOO) where none of the hg V and U5b1b1a lineages distinctive of the Saami could be detected. We show here that the mitochondrial ancestors of the Saami could not be identified in the ancient NEE populations of aUzPo or aBOO, despite the latter site being within the area occupied by Saami today. The widespread modern-day distribution of U5b1 and V lineages makes it difficult to identify the origins of the Saami [32]. Sub-haplogroup U5b1b1 to which the ‘Saami motif’ belongs was proposed to have originated and spread from southern/central Europe after the Late Glacial Maximum [32]–[33]. Despite its clear association with Saami ancestry, the ‘Saami motif’ also occurs at low frequency (below 1%) in a wide range of non-Saami populations in Europe, and haplotypes closely related to the ‘Saami motif’ have even been found in modern Berbers of North Africa [33]. Two origins have been proposed on the basis of archaeological and genetic evidence [24], [32]. First, ancestors of the Saami were suggested to have reached Fennoscandia from Western Europe along the Atlantic cast of Norway as part of the expansion of Mesolithic post-Ahrensburgian cultures (Fosna-Hensbacka and Komsa) in the early Holocene (∼10,000–11,000 yBP). Alternatively, the Saami were proposed to find their origins in Mesolithic post-Swiderian cultures (Kunda, Veretye, Suomusjärvi), which had moved from Poland into NEE also in the early Holocene [24]. The data from aUzPo, in which neither U5b1 or V could be detected, does not support the latter hypothesis. If migrations brought U5b1 and V to Fennoscandia from the East, they must have occurred after 7,500 yBP or have had a weak genetic impact on surrounding populations of NEE. Saami mtDNA diversity has been influenced by a combination of founder event(s), (multiple) bottlenecks, and reproductive isolation, which are likely due to the challenging conditions of life in the subarctic taiga/tundra [32]. The complex demographic history of Saami renders their population history difficult to reconstruct on the basis of modern genetic data alone. Further temporal population samples will be required, especially along the proposed alternative western migration route into sub-arctic Europe.

Individuals from 7,500 year-old aUzPo and 3,500 year-old aBOO show remarkable genetic dissimilarities with present-day North East Europeans: high frequencies of hg U, the presence of mtDNA lineages of ‘Central/East Siberian’ origin, and near absence (one out of 34 samples) of hg H which comprises up to ∼50% in extant European populations [5]. The results of our coalescent simulation analyses show that the models that take account of genetic input(s) from CE are better supported and could explain the genetic discontinuity observed between either aUzPo or aBOO and the modern population of NEE (Figure 5). The mtDNA lineages with a clear Central/Western European signature and currently prevalent in NEE might have reached the western Baltic and southern Scandinavia during the continuing influx of farming populations from Central or lastly southeastern Europe [13], [58], as from 6,000 yBP onwards [71]–[74]. However, intruding Neolithic farmers never reached Karelia and Fennoscandia [75], so the change in population would have to be a post-Neolithic process or to be due to migrations from other sources. The major prehistoric migration in this area was associated with the spread of early pottery from the East into the Baltic, Karelia and Fennoscandia starting around 7,000 yBP. This migration might have contributed to an early population change in Karelia and Fennoscandia as well, but the mtDNA characteristics of the populations involved is presently unknown [76]–[78]. As for Siberia, a general push-back of populations by an expansion of populations from the South-West is discussed [62]. Thus, the present-day distribution of populations similar to aUzPo and aBOO might just be a remnant of a once much larger extension across western and Central northern Eurasia, which is consistent with frequencies of hgs U4 and U5, i.e. the Palaeolithic/Mesolithic genetic substratum, have remained higher in extant populations of NEE, the VUB and Western Siberia than in central Europeans, where these were largely replaced at the onset of the Neolithic [16], [79]. Genetic discontinuity between aUzPo, aBOO and present-day populations of NEE was also observed at the haplotype level, as seen by the lack of matches between lineages from ancient individuals and from present-day NEE (e.g., ‘Central/East Siberian’ lineages in aBOO), or by their total absence in all Eurasian populations of the comparative dataset. A good example is the haplotype C1 found in aUzPo, which is absent in modern-day Eurasians and in all other foraging populations of Europe. This indicates that hg C1 was rare and probably preserved in aUzPo by a relative reproductive isolation, previously proposed for Mesolithic hunter-gatherers of NEE on the basis of odontometric [41] and craniometric [80] analyses. These results do not exclude a common origin for European foragers but highlight differentiating consequences of post-glacial founder effects followed by reproductive isolation among Palaeolithic/Mesolithic groups. Genetic discontinuity between prehistoric populations of Europe may have been caused by the random loss of genetic diversity through drift, which is likely to have been accelerated in small and isolated groups, such as aUzPo and aBOO. In the Kola Peninsula, the scarcity in the archaeological records observed in the Kola Peninsula for the Early Metal Age was interpreted as an indication of drastic size reductions of human groups, as a response to deteriorating climatic conditions ∼2,500 yBP [24]. This could have lead to the local extinction of mtDNA lineages of Siberian origin detected in aBOO in the Kola Peninsula.

Overall, our results illustrate the power of aDNA to reconstruct the complex genetic history of NEE, which is made of past migrations from both Siberia and Europe. Ancient DNA also reveals the plasticity of demographic events in human populations at both the scale of NEE and Eurasia. Future accumulation of genetic data from ancient populations will make it possible to establish more genetic relationships between past human populations in space and time.

## Materials and Methods

### Sample description and archaeological context

A total of 146 human teeth—representing 74 individuals—were collected from three archaeological sites in northwestern Russia: Yuzhnyy Oleni Ostrov, Popovo, and Bolshoy Oleni Ostrov (under custody of the Kunstkamera Museum, St Petersburg, Russia; Figure S1, Table S2).

The oldest samples were collected in the Mesolithic graveyards of Yuzhnyy Oleni Ostrov (aUz; ‘Southern Reindeer Island’ in Russian) and Popovo (aPo). Ninety-six teeth representing 48 individuals were obtained from the Yuzhnyy Oleni Ostrov archaeological site, which is located on Yuzhnyy Oleni Island, Onega Lake, Karelia (61°30′N 35°45′E). The site was first discovered in the 1920s during quarrying excavations, which led to the subsequent destruction of most parts of the graveyard. Scientific excavation of the site by Soviet archaeologists in the 1930s and the 1950s eventually unearthed a total of 177 individuals in 141 different mortuary features [81]. The population size of the burial ground before its partial destruction was estimated at around 500 individuals [82]. The Yuzhnyy Oleni Ostrov graveyard stands out from other Mesolithic sites in Europe by its abundance and diversity of mortuary features. First identified as a Neolithic graveyard, a later reanalysis and radiocarbon dating revealed an age of around 7,000–7,500 uncal. yBP [83]. For Popovo, 6 teeth belonging to 3 individuals were obtained from the archaeological site located on the bank of the Kinema River, in the Archangelsk region (64°32′N 40°32′E). The wide range of dates obtained for this site (9,000–9,500 uncal. yBP and 7,500–8,000 uncal. yBP [84]). We expect that the radiocarbon dates for both the sites of Popovo and Yuzhnyy Oleni Ostrov will be revised, as potential freshwater-derived reservoir effects impacting the dates are currently investigated (T. Higham, personal communication). The sites of aUz and aPo are located along one of the proposed eastern routes for the introduction of Saami-specific mtDNA lineages [32]. Results from odontometric analyses suggested a direct genetic continuity between the Mesolithic population of Yuzhnyy Oleni Ostrov and present-day Saami [41]. Due to the small sample size, and the temporal and geographic proximity of aPo and aUz, the specimens from these sites were pooled for statistical analyses (aUzPo).

We also analyzed human remains from the Early Metal Age archaeological site of Bol'shoy Oleni Ostrov (aBOO; ‘Great Reindeer island’ in Russian) in the Kola Peninsula. This site is located within the area currently inhabited by Saami individuals. Fourty-five teeth representing 23 individuals were obtained from this archaeological site, located in the Murmansk region, Kola Peninsula (68°58′N 33°05′E). Several excavation campaigns have been undertaken between 1927 and 2006. Radiocarbon dates for two graves were obtained from the Oxford Radiocarbon Accelerator Unit, United Kingdom, and revealed an age of around 3237±32 yBP (calibrated dates in years before 1950, 3525–3440 BC (68.2%) and 3610–3420 BC (95.4%)) and 3195±39 yBP; calibrated dates, 3500–3430 BC (68.2%) and 3530–3390 BC (95.4%)) for grave 12 and grave 13, respectively, corresponding to the Early Metal Age. The organic preservation of artifacts made of bone, antlers and wood in this site is exceptional for this time period and geographical location [42].

### Ancient DNA work

DNA isolation, amplification and quantitation were performed at the aDNA laboratory of the Australian Centre for Ancient DNA (ACAD), University of Adelaide. Whenever possible, two distinct teeth were analyzed for each ancient individual. The outer surface of each tooth was decontaminated, first, through exposure to ultra-violet (UV) light for 20 min on each side, then, through gentle wiping using a paper towel soaked in sodium hypochlorite (bleach). The protocol described in [85] was followed to isolate DNA from powdered teeth. Given the archaeological and anthropological value of the samples from aUz, aPo and aBOO, their morphological integrity had to be maintained: tooth powder was collected by cutting off the crown of each tooth and drilling inside the root using a dental drill at low speed. Collecting material from only the dental pulp and dentin may prevent the risk of contamination by exogenous DNA, as the inside of the teeth may be protected from the environment by the enamel.

The mtDNA HVR-I was amplified and sequenced between np 16056 and 16410 as described in [85]. The GenoCore22 reaction described in [85] was used to type 22 haplogroup-diagnostic SNPs in the mtDNA coding-region (Table S3). Twenty-two fragments of mtDNA were amplified simultaneously in a multiplex reaction and SNPs were detected using Single-Base Extension (SNaPshot kit, Applied Biosystems).

The copy-number of two HVR-I fragments - L16209/H16303 (133 bp) and L16209/H16348 (179 bp) - was estimated in selected aDNA extracts (individuals UZOO-43, UZOO-79, BOO72-1, and BOO72-9) by quantitative real-time PCR following the protocol detailed in [16] (Table S4).

Six individuals were randomly selected (UZOO-77, BOO57-1, BOO72-1, BOO72-4, BOO72-7, and BOO72-15), for which the second sample was sent to G.B. at the Johannes Gutenberg University of Mainz for independent replication of DNA extraction, HVR-I amplification and direct sequencing. PCR products were cloned and sequenced. Ancient DNA work at the Johannes Gutenberg University was carried out according to protocols described in [85].

### Authentication of the mtDNA data

Strict precautions were taken in order to minimize the risk of contamination by modern DNA and detect artefactual mutations arising from contamination and aDNA degradation. Seven criteria support the authenticity of the mtDNA data presented here.

1. Pre-PCR DNA work was carried out at the ACAD, a purpose-built laboratory dedicated to aDNA studies. The laboratory is under positive air-pressure and physically isolated from any molecular biology laboratory amplifying DNA. Routine decontamination of the laboratory surfaces and instruments involves exposure to UV radiation and thorough cleaning using bleach, decon90 (decon) and ethanol. In order to protect the laboratory environment from modern human DNA, researchers are required to wear protective clothes consisting of a whole body suit, a face-mask, a face-shield, gumboots, and three pairs of surgical gloves that are changed between individual working steps.
2. Blank controls (one extraction blank for every five ancient samples and two PCR/GenoCoRe22 blank controls for every six reactions) allowed monitoring and controlling large-scale and systematic contamination within the laboratory or in the reagents. In addition, haplotypes similar to those of the users of the laboratory could not be observed from aDNA extracts. Mitochondrial DNA data from the archaeologists and anthropologists involved in the collection of the samples was not available. However, we estimate as rather low the probability that contamination by a few modern-day individuals would generate the diversity and specific patterns of mtDNA lineage distribution observed in the ancient populations under investigation.
3. Multiple replications of HVR-I amplification and direct sequencing were performed in order to detect artefactual sequences due to contamination, DNA degradation or jumping PCR events. When possible, two teeth were collected for each individual and DNA was extracted independently from each sample (i.e., a minimum of two extractions per individual). For each extract, each PCR fragment and each GenoCore22 SNP position was genotyped from at least two independent PCR products (i.e., a minimum of four independent PCRs per fragment and four GenoCoRe22 reactions per individual). This strategy was chosen over cloning for most of the individuals examined here. In low-template conditions, clone sequences can represent the small population of highly degraded starting DNA templates that were exponentially amplified by the one single PCR. In our opinion, a hierarchical replication strategy based on multiple independent amplifications is a powerful alternative to cloning in order to detect artefactual mutations and provides confidence about the authenticity of our DNA sequences.
4. The independent replications of DNA extraction/amplification/direct sequencing carried out at the Johannes Gutenberg University confirmed the diagnostic mutations initially identified at the ACAD in the six selected individuals: UZOO-77, BOO57-1, BOO72-1, BOO72-4, BOO72-7, and BOO72-15 (Figure S3).
5. Sequencing of cloned PCR products for six individuals (individuals UZOO-77, BOO57-1, BOO72-1, BOO72-4, BOO72-7, and BOO72-15) allowed the corresponding haplotypes to be verified. The sequences showed nucleotide positions modified by post-mortem damage as inconsistent cytosine to thymine or guanine to adenosine base changes (Figure S3). For one individual (BOO57-1), independent replications and cloning did not allow allelic resolution at np 16390R. At this position, double peaks (A/G) were observed in direct sequencing, and alleles A and G showed an equal distribution among clones (Figure S3B). This position might be heteroplasmic in the BOO57-1 individual, as np 16390 has been described as a mutational hotspot [86], and therefore might as well be a hotspot for post-mortem DNA damage exhibiting a high rate of post-mortem cytosine deamination.
6. The amount of template mtDNA molecules for two fragments of different sizes (133 bp and 179 bp) was estimated and compared in order to test whether they were consistent with low concentrations of recent human mtDNA contaminants in six selected aDNA extracts (UZOO43, UZOO74, BOO72-1, BOO79-9, and two ancient co-extracts from a related study; data not shown). The size distribution of endogenous aDNA molecules was previously shown to be skewed towards smaller fragment sizes due to post-mortem damage, i.e. DNA fragmentation [87]–[90]. Here, the Shapiro-Wilk W test was first used to verify that the number of copies for each fragment followed a normal distribution (*p* = 0.2215 for the 133 bp short fragment and *p* = 0.5381 for the 179 bp long fragment). A significantly larger number of copies for the shorter (133 bp) compared to the larger (179 bp) fragment was statistically confirmed by a one-tailed paired t-test (*p* = 0.04337) in R version 2.12 (R Development Core Team, http://www.R-project.org). Quantitative PCR results suggest a low level of contaminating DNA molecules, the presence of which would have been detected by higher copy-number of longer (less fragmented) DNA molecules.
7. The phylogenetic consistency of the haplotypes and matching hgs assignments of both HVR-I data and coding region SNPs, were indicative of the robustness of the mtDNA typing approach presented here.

### Populations used in comparative analyses

Mitochondrial DNA data from aUzPo and aBOO were compared to data obtained from other ancient and present-day populations. Data for extant populations were compiled in the MURKA mtDNA database and integrated software, which currently contains 168,000 HVR-I records from published studies and is curated by co-authors V. Z., O.B. and E.B. of the Russian Academy of Medical Sciences, Moscow. A sub-sample of 91 ancient and modern Eurasian populations (∼28,652 individuals) was used for comparative analysis. Names of modern-day populations were abbreviated using ISO codes in capital letters, and in small letters when ISO codes were not available. Unless specified otherwise, the same population labels were used for all the maps and analyses in this study, i.e., PCA, haplotype sharing and analysis of coalescent simulations (Table S5).

### Principal Component Analysis

PCA was performed using the hg frequency database for ancient and modern-day populations described in Table S5. We used a total of 19 variables to perform the PCA. Seventeen of these variables were frequencies of hgs C, D, H, HV, I, J, K, N1, T, U2, U4, U5a, U5b, V, W, X, and Z. In addition, the frequencies of six ‘east Eurasian’ hgs were pooled into one ‘EAS’ group including hgs A, B, E, F, G, and Y. Finally, frequencies of eight hgs found at lower frequencies in Eurasia were pooled into the ‘misc’ group including hgs L, M*, N*, U1, U6, U7, U8. By pooling and removing rare hgs (with frequencies below 1%) we could reduce statistical noise. In order to assess the impact of potential maternal kinship within the sites of aUzPo and aBOO, we performed an additional PCA, in which redundant haplotypes, i.e. haplotypes found in more than one individual at a given site, were counted only once (Figure S2A and Figure S2B). PCA was carried out using a customized script based on the function *prcomp* in R version 2.12 (R Development Core Team, http://www.R-project.org).

### Genetic distance mapping

The genetic distances between 144 pools of extant Eurasian populations and each of the aUzPo and aBOO populations were calculated using the software DJ (written by Yuri Seryogin, and freely available at http://genofond.ru, also see [29]). The software GeneGeo written by S.K. was used to plot genetic distances onto geographic maps (as described in [16]).

### Haplotype sharing analysis

In haplotype sharing analyses, we calculated the percentages of shared haplotypes between 29 extant populations and the ancient populations of aUzPo and aBOO. A database of mtDNA haplotypes was collated for modern-day populations, each containing 500 individuals. We pooled populations of less than 500 individuals on the basis of their geographical and/or linguistic similarities. For extant populations of more than 500 individuals, we randomly sub-sampled 500 individuals from the populations. For each haplotype of aUzPo and aBOO, we counted the number of haplotype matches found in each of the extant populations of the comparative database. This number was divided by the sample size in order to obtain the percentage of shared haplotypes. The same procedure was applied to calculate the percentage of shared haplotypes between the ancient populations studied here (aUzPo and aBOO) and previously described ancient populations. Percentages of shared haplotypes between ancient and present-day populations were represented in a bar plot. Percentages of shared haplotypes among ancient populations were represented in a table, the cells of which were colored according to a gradient reflecting the haplotypic similarities between the populations compared.

### Coalescent simulations

In coalescent simulation analyses we considered the ancient populations of aUzPo, aBOO, Central/East/Scandinavian European hunter-gatherers (aHG [12], [14], aPWC [13]), and the modern populations of NEE, CE, and Saami (saa). Population statistics (haplotype diversity and fixation indexes, F_ST_) for the ancient and extant populations were calculated in Arlequin version 3.11 (Table 2, [91]).

In BayeSSC [49], genealogies were simulated under the following model of sequence evolution: a generation time of 25 years, a mutation rate of 7.5.10^−6^ substitutions/per site/per generation [92], a transition/transversion ratio of 0.9841, and parameters for the gamma distribution of rates along the sequence of 0.205 (theta) and 10 (kappa) [16].

Under the models of genetic continuity H0, the effective population size (N_e_) of a single population was allowed to grow exponentially. The values of the growth rate were drawn from a uniform prior distribution, such that the population has evolved from a Palaeolithic population of N_e_ 5,000 that lived 1,500 generations ago. The values for the modern-day (NEE or saa) N_e_ were drawn from a uniform distribution: we explored present-day N_e_ between 100,000 and 30,000,000 for NEE and 1,000 to 500,000 for saa. Population statistics were estimated at various points in time, corresponding to the age of the ancient populations considered in models H0a to H0e (aUzPo, aBOO, aHG and aPWC).

Under the models of migration H1, we assumed a single NEE population undergoing an exponential growth in N_e_ and being the recipient (sink population) of a migration from CE (source population). Population sizes of each of the present-day sink population (NEE) and source population (CE) were drawn from a uniform distribution of N_e_ varying from 100,000 to 15,000,000 individuals. Migration and divergence times were estimated from uniform distributions (from 2 to 139 generations for migration and from 620 to 2,600 generations for divergence). Three different percentages of the source population size were tested for the value of percentages of migrants: 10%, 50% and 75%.

Population statistics were calculated for 100,000 genealogies simulated using BayeSSC (available at http://www.stanford.edu/group/hadlylab/ssc/index.html). The distribution of six selected population statistics (haplotype diversity and fixation indices F_ST_) were drawn from the simulations and compared to the corresponding observed population statistics in an ABC framework [50], [93]. The 1% of the simulations for which simulated population statistics exhibited the smallest Euclidian distance with observed population statistics was retained to construct posterior distributions of population parameters. From these distributions, values of population parameters that optimized the likelihood of a given model were estimated and used in replacement of priors in demographic models. We finally generated 10,000 genealogies in BayeSSC for these models. BayeSSC outputs were analyzed in R version 2.12 using scripts available on request at http://www.stanford.edu/group/hadlylab/ssc/index.html. Goodness of fit of the different models tested was compared using AICs [51] and Akaike's weights ω [52]–[53] (Table 3).

### Accession numbers

The Genbank accession numbers for the 34 mtDNA sequences reported in this paper are KC414891–KC414924.

## Supporting Information

Figure S1 Pictures of selected samples from Yuzhnyy Oleni Ostrov, Popovo and Bol'shoy Oleni Ostrov. The macroscopic preservation of the selected samples is representative of the general preservation observed in the corresponding sites. Yuzhnyy Oleni Ostrov sample ACAD4719 did not yield reliable mitochondrial hypervariable region I sequences.
(PDF)

Figure S2 Principal Component Analysis of mtDNA haplogroup frequencies, non-redundant ancient haplotypes only. A. Recalculated frequencies. B. PCA plots. The first two dimensions account for 42.4% of the total variance. Grey arrows represent hg loading vectors, i.e., the contribution of each hg. Red dots represent ancient populations described in this study (non-redundant haplotypes only): aUzPo2, Yuzhnyy Oleni Ostrov/Popovo (7,500 uncal. yBP); aBOO2, Bol'shoy Oleni Ostrov (3,500 uncal. yBP). Other ancient populations were labelled as follows: aEG, confederated nomads of the Xiongnu (4,250-2,300 yBP); aHG, Palaeolithic/Mesolithic hunter-gatherers of Central/East Europe (4,250-30,000 yBP); aKAZ, Nomads from Kazakhstan (2,100–3,400 yBP); aKUR, Siberian Kurgans (1,600–3,800 yBP); aLBK, Neolithic individuals from Germany (7,000–7,500 yBP); aLOK, Lokomotiv Kitoi Neolithic individuals (6,130–7,140 yBP); aSP, Neolithic individuals from Spain (5,000–5,500 yBP); aPWC, Scandinavian Pitted-Ware Culture foragers (4,500–5,300 yBP); aUST, Ust'Ida Neolithic population (4,000–5,800 yBP). Extant populations were abbreviated as follows: ALB, Albanians; ale, Aleuts; alt, Altaians; ARM, Armenians; aro, Arorums; AUT, Austrians; AZE, Azerbaijani; BA, Bashkirs; bas, Basques; BEL, Belarusians; BGR, Bulgarians; BIH, Bosnians; BU, Buryats; CHE, Swiss; CHU, Chukchi; CU, Chuvashes; CYP, Cypriots; CZE, Czechs; DEU, Germans; esk, Eskimos; ESP, Spanish; EST, Estonians; eve, Evenks; evn, Evens; FIN, Finns; FRA, French; GBR, British; GEO, Georgians; GRC, Greeks; HRV, Croatians; HUN, Hungarians; ing, Ingrians; IRL, Irish; IRN, Iranians; IRQ, Iraqi; ISL, Icelanders; IT-88, Sardinians; ITA, Italians; JOR, Jordanians; kab, Kabardians; ket, Kets; kham, Khamnigans; khan, Khants; KK, Khakhassians; KO, Komi; kor, Koryaks; KR, Karelians; kur, Kurds; LTU, Lithuanians; LVA, Latvians; man, Mansi; ME, Mari; MNG, Mongolians; MO, Mordvinians; NEN_A, eastern Nenets; NEN_E, western Nenets; nga, Nganasans; niv, Nivkhs; nog, Nogays; NOR, Norwegians; POL, Poles; PRT, Portuguese; PSE, Palestinans; ROU, Romanians; RUS, Russians; SA, Yakuts; saa, Saami; SAU, Saudi Arabians; SE, Ossets; sel, Selkups; sho, Shors; SVK, Slovakians; SVN, Slovenians; SWE, Swedes; SYR, Syrians; TA, Tatars; tel, Telenghits; tof, Tofalars; tub, Tubalars; TUR, Turks; tuv, Tuvinians; UD, Udmurts; UKR, Ukrainians; ulc, Ulchi; vep, Vepses; yuk, Yukaghirs.
(PDF)

Figure S3 Direct and clone sequences for six selected samples. A. UZOO77. B. BOO57-1. C. BOO72-1. D. BOO72-4. E. BOO72-7. F. BOO72-15. “_1” after the individual identification number signifies that the sequences have been obtained after DNA extraction and sequencing from a first sample at the Australian Centre for Ancient DNA. “_2” after the individual identification number signifies that the sequences have been obtained after DNA extraction, cloning and sequencing from a second sample at the Institute of Anthropology, Johannes Gutenberg University of Mainz.
(PDF)

Table S1 Description and references for hg C1 HVR-I sequences found in modern-day populations of Eurasia.
(PDF)

Table S2 Grave and museum collection number for Yuzhnyy Oleni Ostrov, Popovo and Bol'shoy Oleni Ostrov specimens.
(PDF)

Table S3 Results of SNP typing in the mtDNA coding region using the GenoCore22 SNaPshot assay. SNPs typed on the L-strand are reported in capital letters in the reference rCRS profile, whereas SNPs typed on the H-strand are reported in small letters. Missing data signifies allelic dropout or fluorescence signal below the background threshold (100 relative fluorescent units, rfu). ‘g/a’ indicates the presence of a mixed signal for the position interrogated. A mixed signal was repeatedly obtained at position 8994 (haplogroup W) with the detection of an additional G base. However, the rest of the profile never could support phylogenetically the presence of the G base at this particular position. For each individual, profiles were obtained from two independent extracts, except for individual BOO72-9 for which a second samples was not available and for UZOO-77, BOO57-1, BOO72-10, BOO72-4, BOO72-7, BOO72-15, and BOO72-1, for which the second individual was extracted in an independent laboratory. rCRS, revised Cambridge Reference Sequence; hg, haplogroup.
(PDF)

Table S4 Results of quantitative PCR.
(PDF)

Table S5 Details of ancient and modern-day populations used in comparative analyses.
(PDF)
