# Supplementary material for: Ancient DNA Reveals Prehistoric Gene-Flow from Siberia in the Complex Human Population History of North East Europe
Source: PLoS Genet. 2013 Feb 14;9(2):e1003296. doi: 10.1371/journal.pgen.1003296 (PMC3573127; doi:10.1371/journal.pgen.1003296)
Supplement: Figure S1 — Pictures of selected samples from Yuzhnyy Oleni Ostrov, Popovo and Bol'shoy Oleni Ostrov. The macroscopic preservation of the selected samples is representative of the general preservation observed in the corresponding sites. Yuzhnyy Oleni Ostrov sample ACAD4719 did not yield reliable mitochondrial hypervariable region I sequences. (PDF) [file pgen.1003296.s001.pdf]

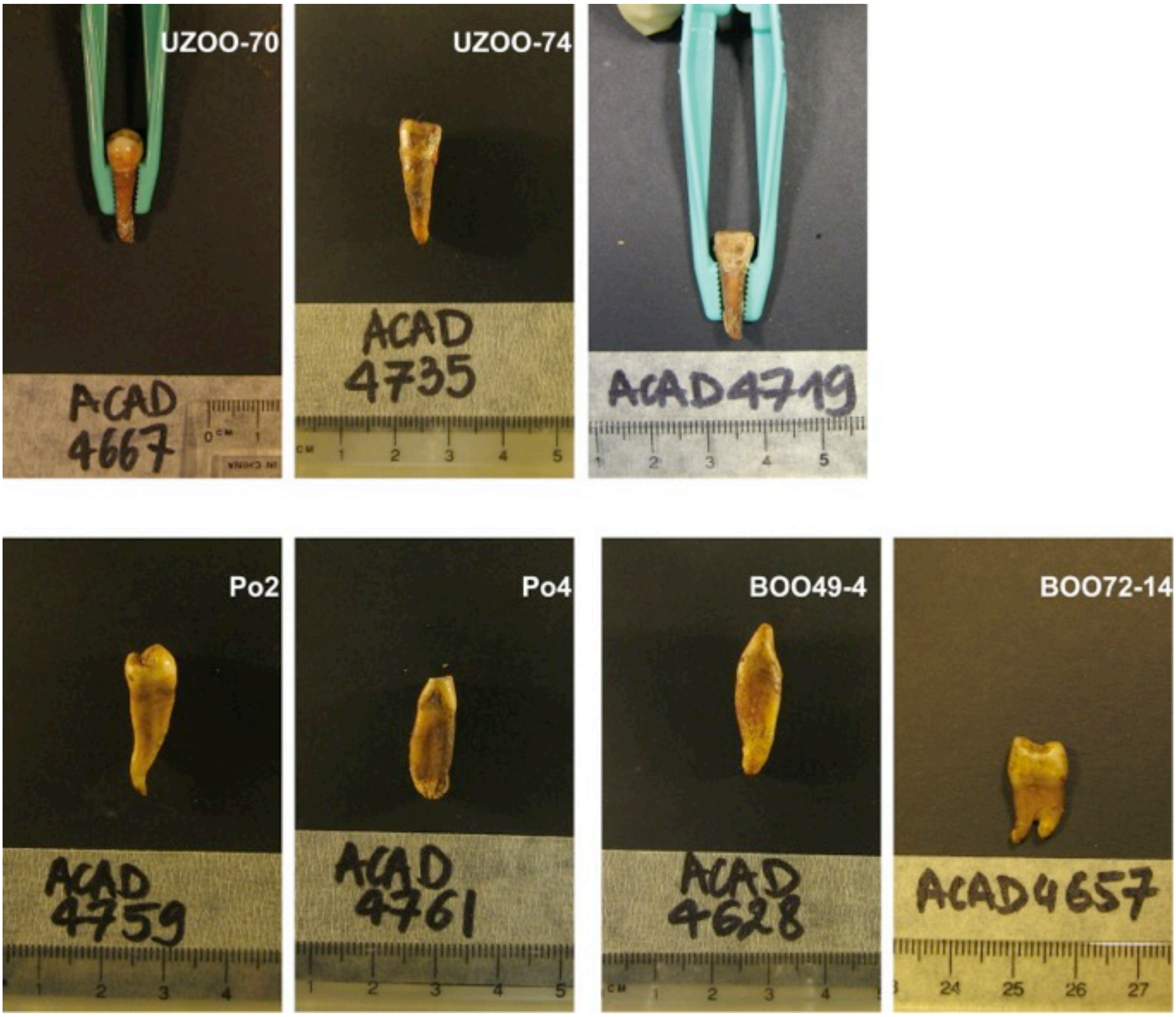

Figure S1. Pictures of selected samples from Yuzhnyy Oleni Ostrov, Popovo and Bol'shoy Oleni Ostrov. The macroscopic preservation of the selected samples is representative of the general preservation observed in the corresponding sites. Yuzhnyy Oleni Ostrov sample ACAD4719 did not yield reliable mitochondrial hypervariable region I sequences.
