# Supplementary material for: Ancient DNA Reveals Prehistoric Gene-Flow from Siberia in the Complex Human Population History of North East Europe
Source: PLoS Genet. 2013 Feb 14;9(2):e1003296. doi: 10.1371/journal.pgen.1003296 (PMC3573127; doi:10.1371/journal.pgen.1003296)
Supplement: Figure S2 — Principal Component Analysis of mtDNA haplogroup frequencies, non-redundant ancient haplotypes only. A. Recalculated frequencies. B. PCA plots. The first two dimensions account for 42.4% of the total variance. Grey arrows represent hg loading vectors, i.e., the contribution of each hg. Red dots represent ancient populations described in this study (non-redundant haplotypes only): aUzPo2, Yuzhnyy Oleni Ostrov/Popovo (7,500 uncal. yBP); aBOO2, Bol'shoy Oleni Ostrov (3,500 uncal. yBP). Other ancient populations were labelled as follows: aEG, confederated nomads of the Xiongnu (4,250-2,300 yBP); aHG, Palaeolithic/Mesolithic hunter-gatherers of Central/East Europe (4,250-30,000 yBP); aKAZ, Nomads from Kazakhstan (2,100–3,400 yBP); aKUR, Siberian Kurgans (1,600–3,800 yBP); aLBK, Neolithic individuals from Germany (7,000–7,500 yBP); aLOK, Lokomotiv Kitoi Neolithic individuals (6,130–7,140 yBP); aSP, Neolithic individuals from Spain (5,000–5,500 yBP); aPWC, Scandinavian Pitted-Ware Culture foragers (4,500–5,300 yBP); aUST, Ust'Ida Neolithic population (4,000–5,800 yBP). Extant populations were abbreviated as follows: ALB, Albanians; ale, Aleuts; alt, Altaians; ARM, Armenians; aro, Arorums; AUT, Austrians; AZE, Azerbaijani; BA, Bashkirs; bas, Basques; BEL, Belarusians; BGR, Bulgarians; BIH, Bosnians; BU, Buryats; CHE, Swiss; CHU, Chukchi; CU, Chuvashes; CYP, Cypriots; CZE, Czechs; DEU, Germans; esk, Eskimos; ESP, Spanish; EST, Estonians; eve, Evenks; evn, Evens; FIN, Finns; FRA, French; GBR, British; GEO, Georgians; GRC, Greeks; HRV, Croatians; HUN, Hungarians; ing, Ingrians; IRL, Irish; IRN, Iranians; IRQ, Iraqi; ISL, Icelanders; IT-88, Sardinians; ITA, Italians; JOR, Jordanians; kab, Kabardians; ket, Kets; kham, Khamnigans; khan, Khants; KK, Khakhassians; KO, Komi; kor, Koryaks; KR, Karelians; kur, Kurds; LTU, Lithuanians; LVA, Latvians; man, Mansi; ME, Mari; MNG, Mongolians; MO, Mordvinians; NEN_A, eastern Nenets; NEN_E, western Nenets; nga, Nganasans; niv, Nivkhs; nog, N [file pgen.1003296.s002.pdf]

A

Population	Hg frequency
**aUzPo2**	
U2	14.3%
U4	42.8%
U5a	14.3%
H	14.3%
C	14.3%
**aBOO2**	
U4	11.1%
U5a	22.2%
T	11.1%
C	22.2%
D	11.1%
Z	22.2%

B

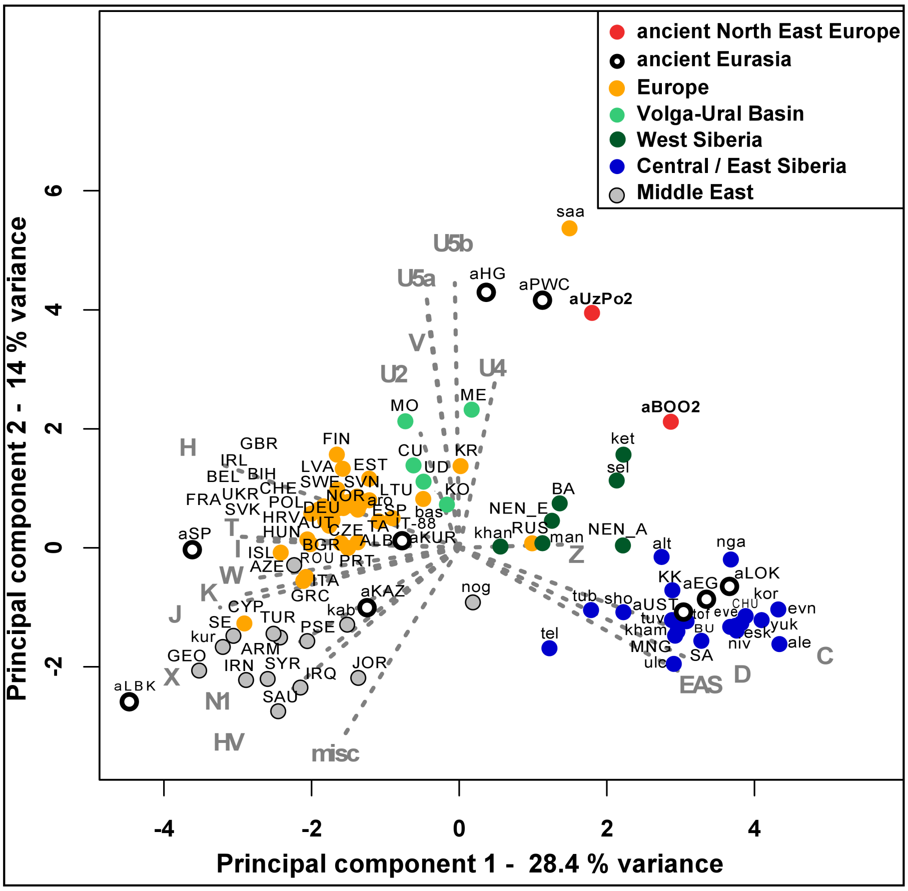

Figure S2. Principal Component Analysis of mtDNA haplogroup frequencies, non-redundant ancient haplotypes only. A. Recalculated frequencies. B. PCA plot. The first two dimensions account for 42.4% of the total variance. Grey arrows represent hg loading vectors, i.e., the contribution of each hg. Red dots represent ancient populations described in this study (non-redundant haplotypes only): aUzPo2, Yuzhnyy Oleni Ostrov/Popovo (7,500 uncal. yBP); aBOO2, Bol'shoy Oleni Ostrov (3,500 uncal. yBP). Other ancient populations were labelled as follows: aEG, confederated nomads of the Xiongnu (4,250-2,300 yBP); aHG, Palaeolithic/Mesolithic hunter-gatherers of Central/East Europe (4,250-30,000 yBP); aKAZ, Nomads from Kazakhstan (2,100-3,400 yBP); aKUR, Siberian Kurgans (1,600-3,800 yBP); aLBK, Neolithic individuals from Germany (7,000-7,500yBP); aLOK, Lokomotiv Kitoi Neolithic individuals (6,130-7,140 yBP); aSP, Neolithic individuals from Spain (5,000-5,500 yBP); aPWC, Scandinavian Pitted-Ware Culture foragers (4,500-5,300 yBP); aUST, Ust'Ida Neolithic population (4,000-5,800 yBP). Extant populations were abbreviated as follows: ALB, Albanians; ale, Aleuts; alt, Altaians; ARM, Armenians; aro, Arorums; AUT, Austrians; AZE, Azerbaijani; BA, Bashkirs; bas, Basques; BEL, Belarusians; BGR, Bulgarians; BIH, Bosnians; BU, Buryats; CHE, Swiss; CHU, Chukchi; CU, Chuvashes; CYP, Cypriots; CZE, Czechs; DEU, Germans; esk, Eskimos; ESP, Spanish; EST, Estonians; eve, Evenks; evn, Evens; FIN, Finns; FRA, French; GBR, British; GEO, Georgians; GRC, Greeks; HRV, Croatians; HUN, Hungarians; ing, Ingrians; IRL, Irish; IRN, Iranians; IRQ, Iraqi; ISL, Icelanders; IT-88, Sardinians; ITA, Italians; JOR, Jordanians; kab, Kabardians; ket, Kets; kham, Khamnigans; khan, Khants; KK, Khakhassians; KO, Komi; kor, Koryaks; KR, Karelians; kur, Kurds; LTU, Lithuanians; LVA, Latvians; man, Mansi; ME, Mari; MNG, Mongolians; MO, Mordvinians; NEN_A, eastern Nenets; NEN_E, western Nenets; nga, Nganasans; niv, Nivkhs; nog, Nogays; NOR, Norwegians; POL, Poles; PRT, Portuguese; PSE, Palestinians; ROU, Romanians; RUS, Russians; SA, Yakuts; saa, Saami; SAU, Saudi Arabians; SE, Ossets; sel, Selkups; sho, Shors; SVK, Slovakians; SVN, Slovenians; SWE, Swedes; SYR, Syrians; TA, Tatars; tel, Telenghits; tof, Tofalars; tub, Tubalars; TUR, Turks; tuv, Tuvinians; UD, Udmurts; UKR, Ukrainians; ulc, Ulchi; vep, Vepses; yuk, Yukaghirs.
