# Supplementary material for: Ancient DNA Reveals Prehistoric Gene-Flow from Siberia in the Complex Human Population History of North East Europe
Source: PLoS Genet. 2013 Feb 14;9(2):e1003296. doi: 10.1371/journal.pgen.1003296 (PMC3573127; doi:10.1371/journal.pgen.1003296)
Supplement: Figure S3 — Direct and clone sequences for six selected samples. A. UZOO77. B. BOO57-1. C. BOO72-1. D. BOO72-4. E. BOO72-7. F. BOO72-15. “_1” after the individual identification number signifies that the sequences have been obtained after DNA extraction and sequencing from a first sample at the Australian Centre for Ancient DNA. “_2” after the individual identification number signifies that the sequences have been obtained after DNA extraction, cloning and sequencing from a second sample at the Institute of Anthropology, Johannes Gutenberg University of Mainz. (PDF) [file pgen.1003296.s003.pdf]

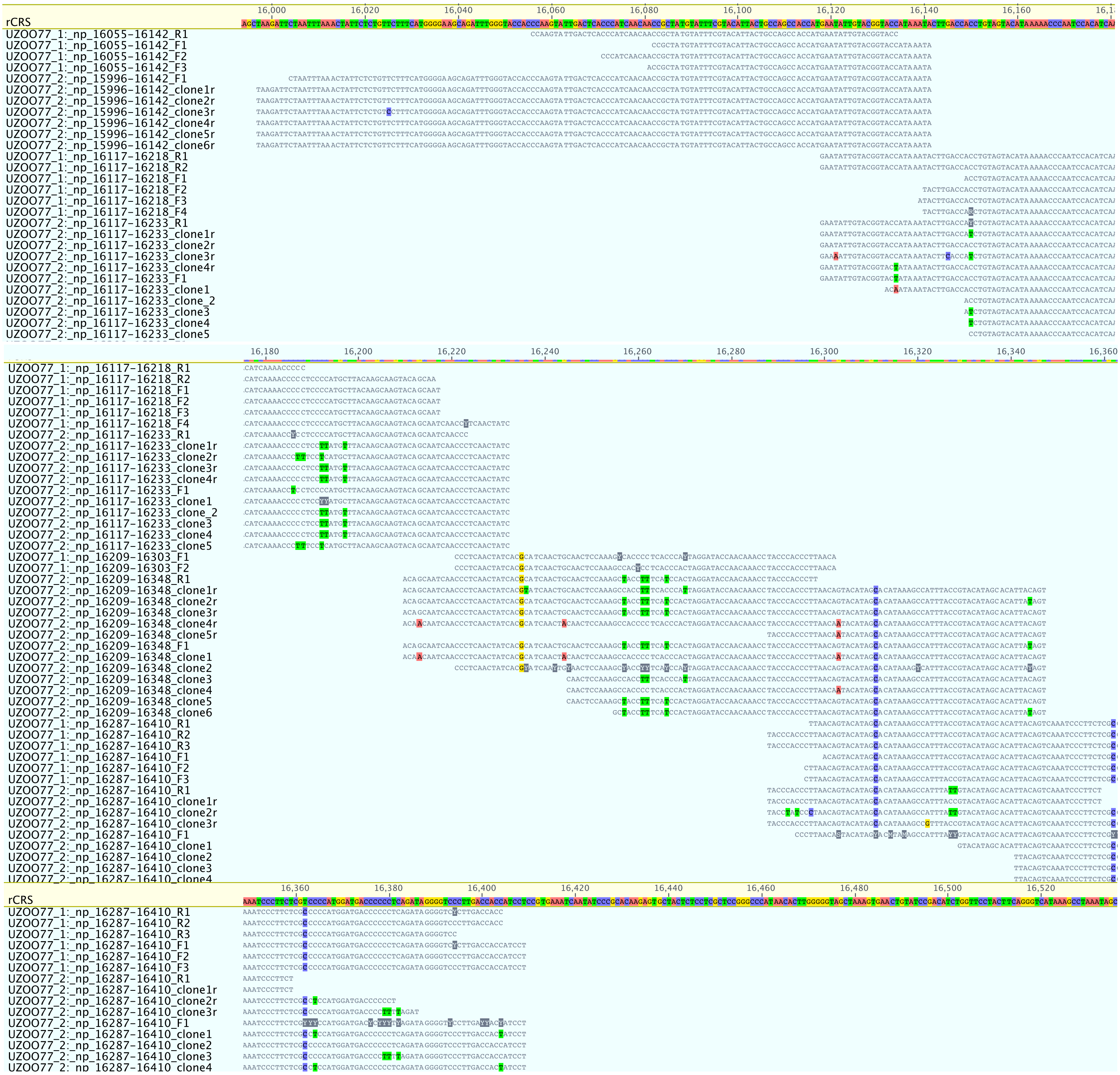

A- Direct sequences and clone sequences for Yuzhnyy Oleni Ostrov individual UZOO77

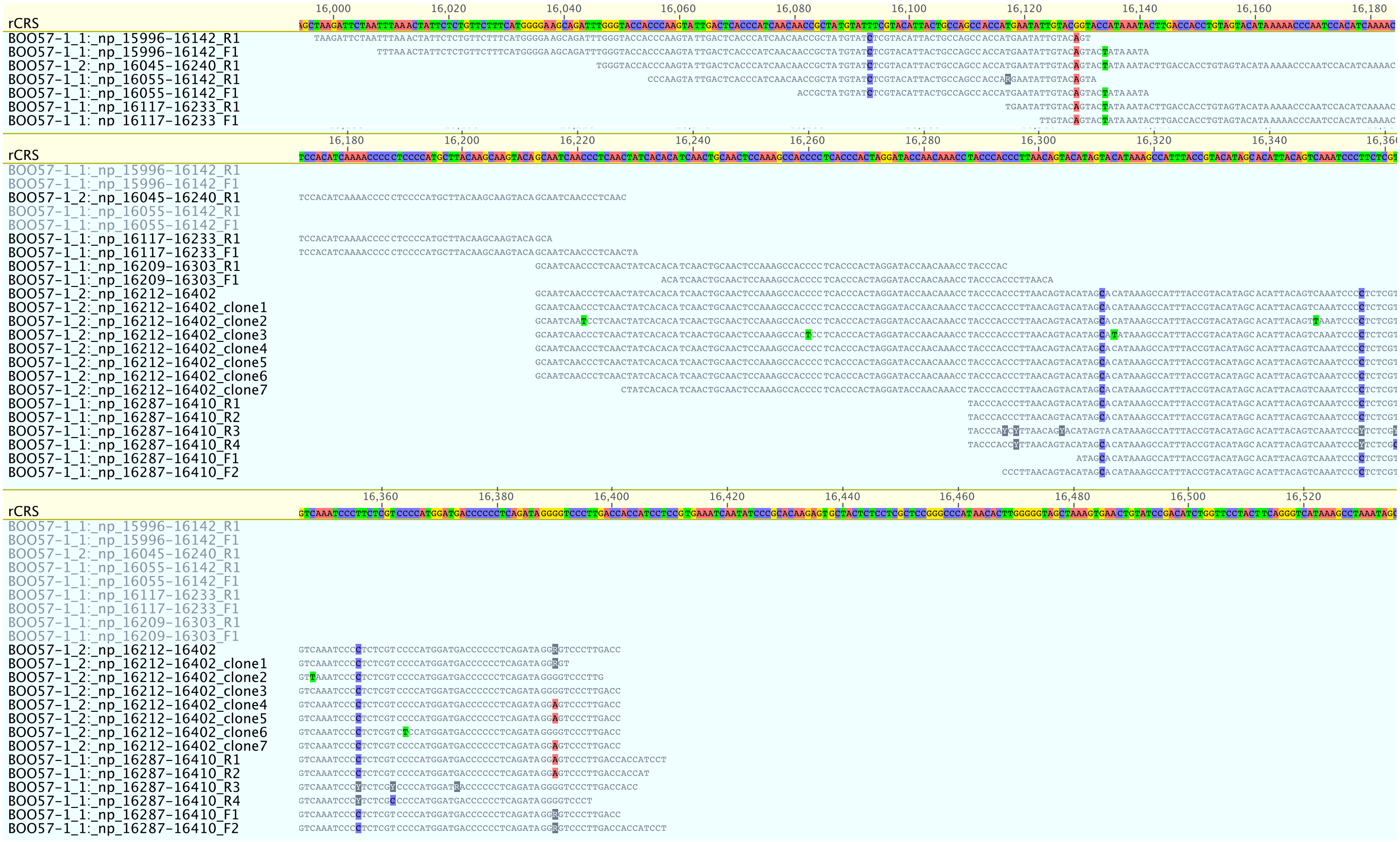


B- Direct sequences and clone sequences for Bol'shoy Oleni Ostrov individual BOO57-1

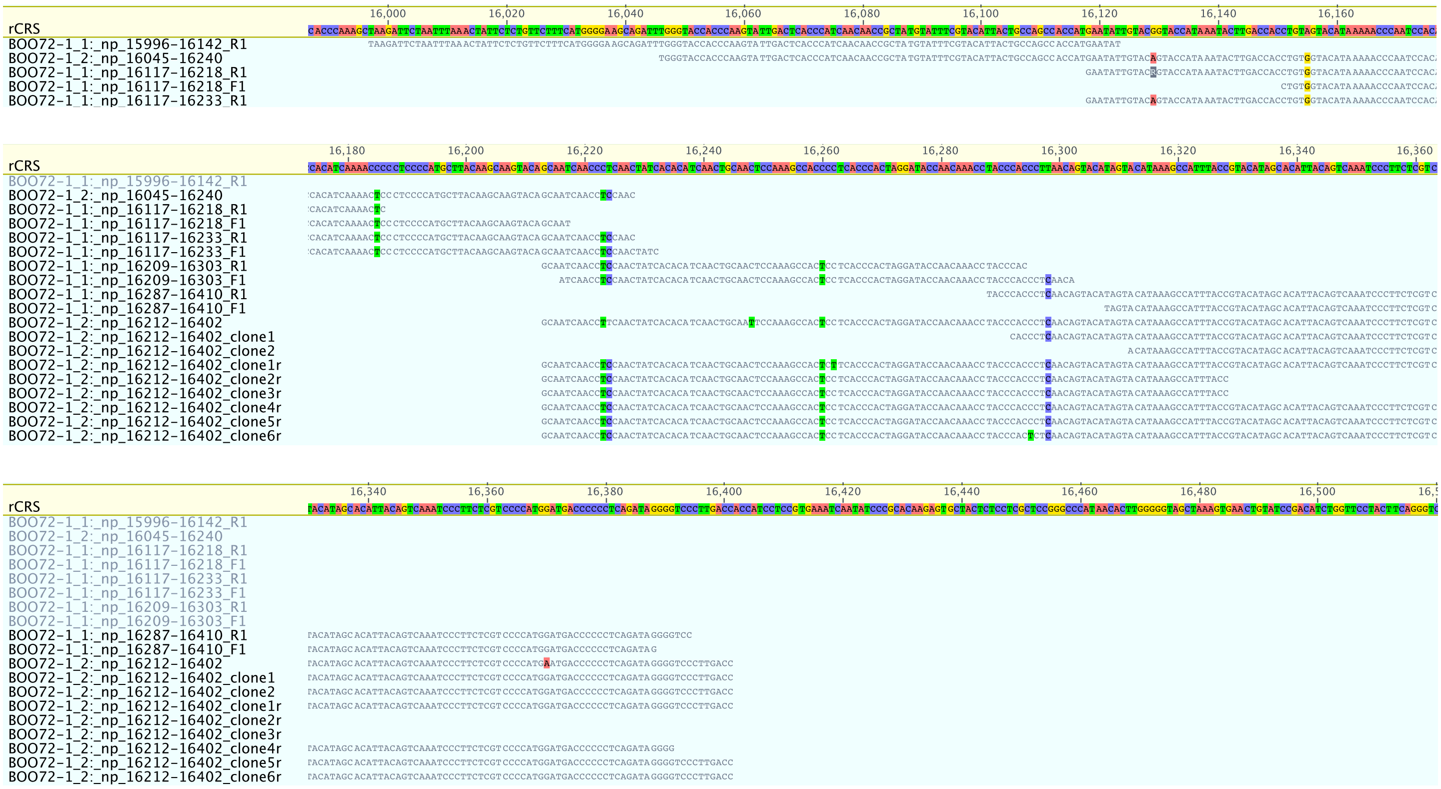


C- Direct sequences and clone sequences for Bol'shoy Oleni Ostrov individual BOO72-1

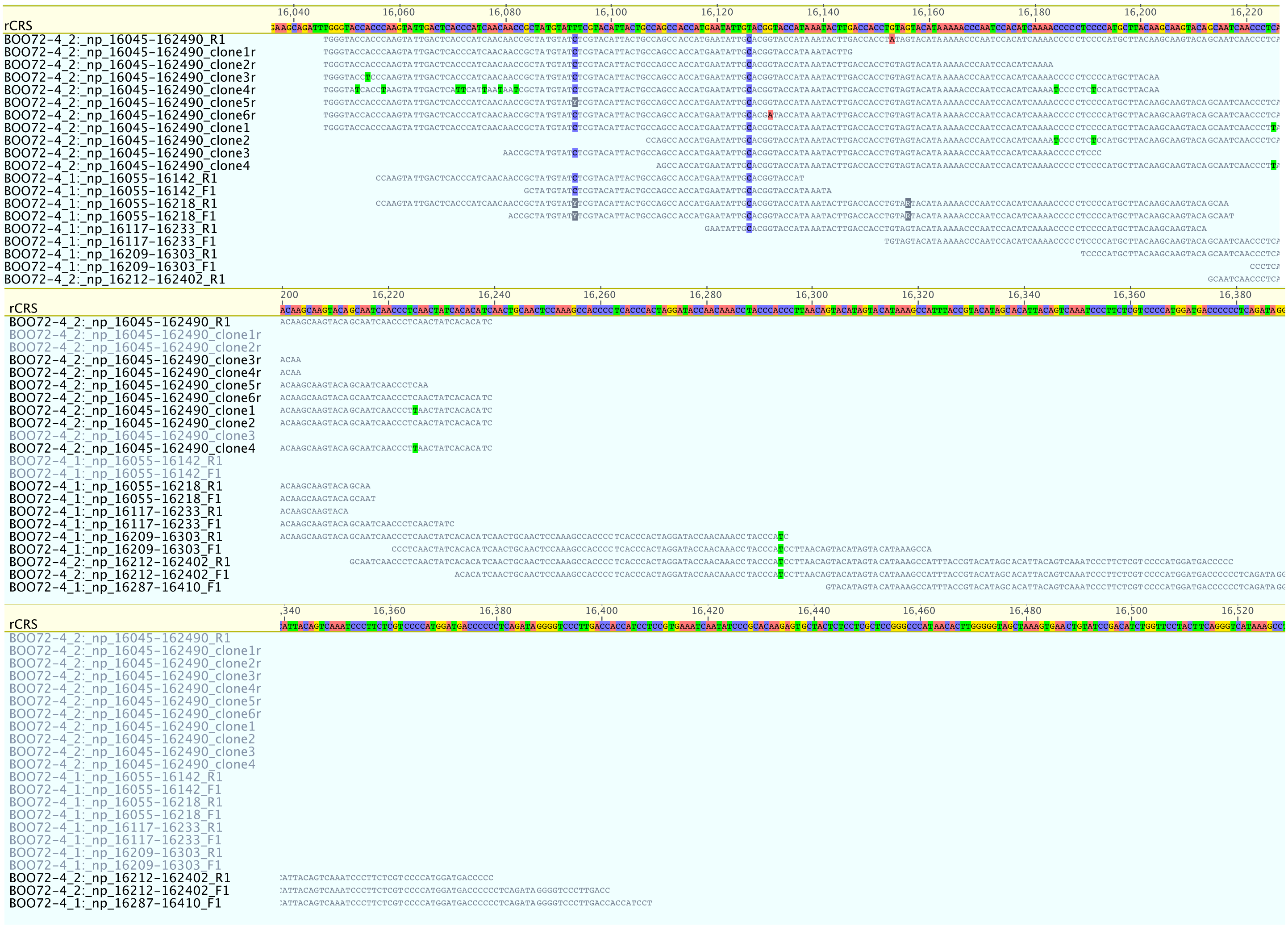


D- Direct sequences and clone sequences for Bol’shoy Oleni Ostrov individual BOO72-4

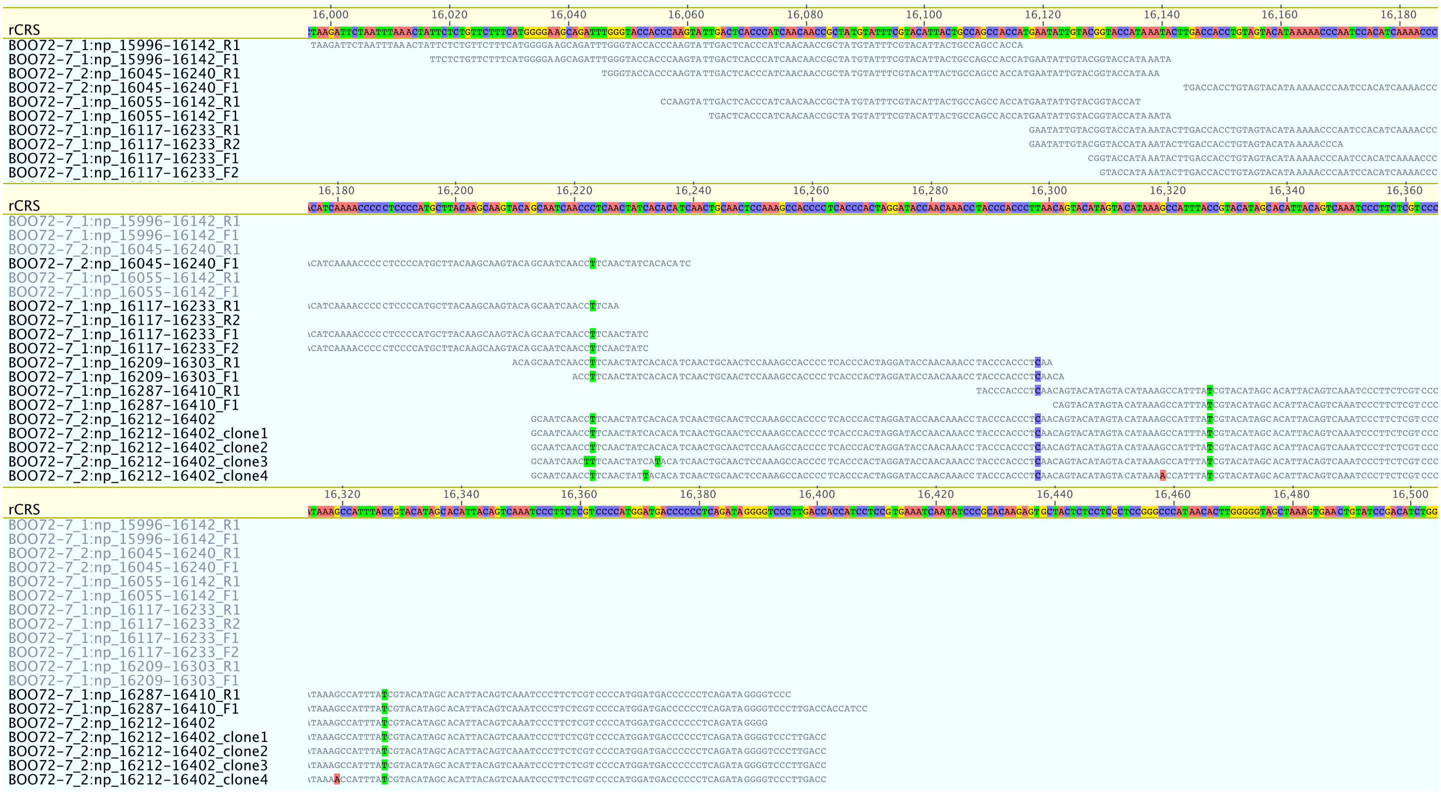


E- Direct sequences and clone sequences for Bol’shoy Oleni Ostrov individual BOO72-7

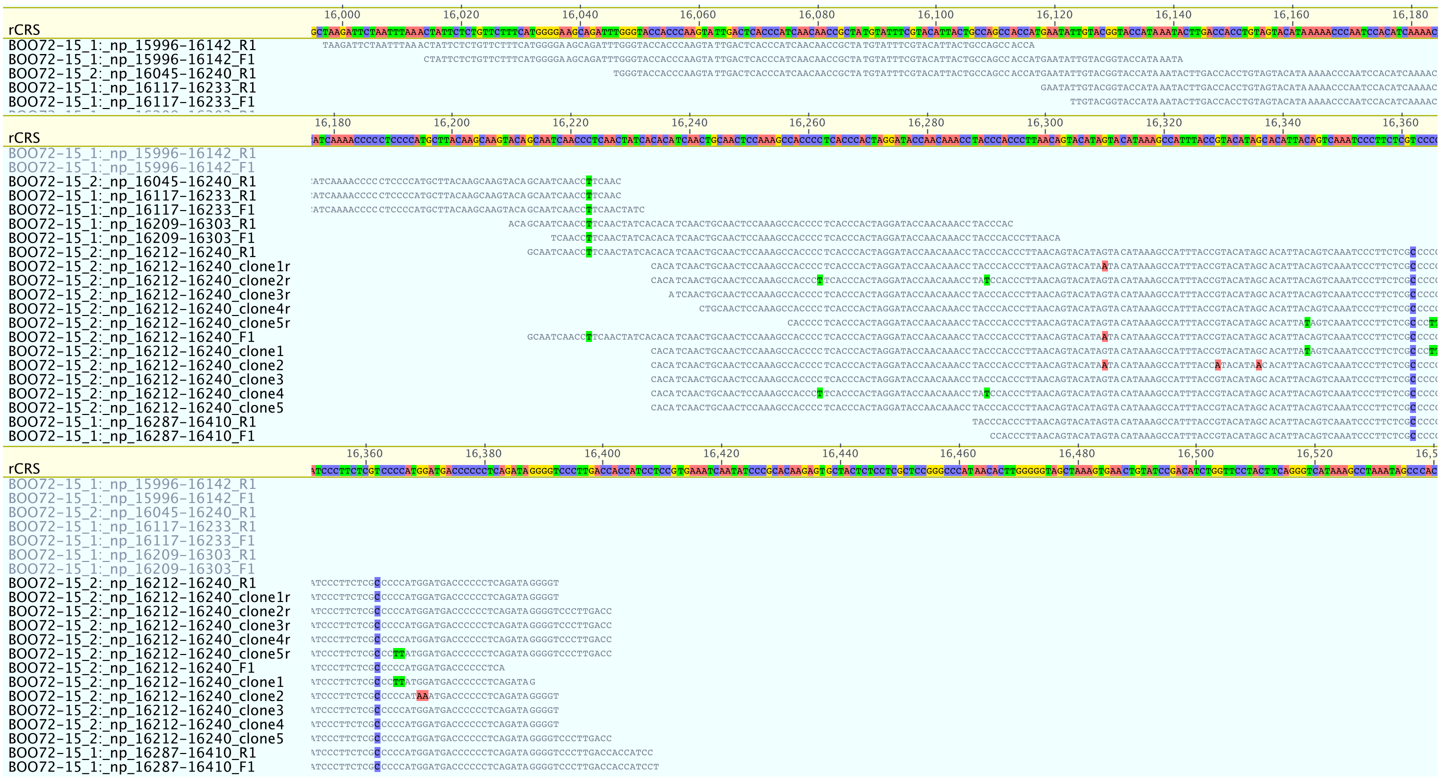


F- Direct sequences and clone sequences for Bol'shoy Oleni Ostrov individual BOO72-15

**Figure S3. Direct and clone sequences for six selected samples.** A. UZOO77. B. BOO57-1. C. BOO72-1. D. BOO72-4. E. BOO72-7. F. BOO72-15. "_1" after the individual identification number signifies that the sequences have been obtained after DNA extraction and sequencing from a first sample at the Australian Centre for Ancient DNA. "_2" after the individual identification number signifies that the sequences have been obtained after DNA extraction, cloning and sequencing from a second sample at the Institute of Anthropology, Johannes Gutenberg University of Mainz.
