# Supplementary material for: Ancient DNA Reveals Prehistoric Gene-Flow from Siberia in the Complex Human Population History of North East Europe
Source: PLoS Genet. 2013 Feb 14;9(2):e1003296. doi: 10.1371/journal.pgen.1003296 (PMC3573127; doi:10.1371/journal.pgen.1003296)
Supplement: Table S1 — Description and references for hg C1 HVR-I sequences found in modern-day populations of Eurasia. (PDF) [file pgen.1003296.s004.pdf]

Table S1. Description and references for hg C1 HVR-I sequences found in modern-day populations of Eurasia.

C1 clade	Geographic /Ethnic origin	HVR-I sequence (np 16056-16409), Minus np 16000	Reference
C1?	Lebanese	223T-266T-298C-325C-327T	[1]
C1?	Lebanese	223T-266T-298C-325C-327T	[1]
C1?	Bashkir	223T-298C-325C-327T-362C	[2]
C1?	Bashkir	223T-298C-325C-327T-362C	[2]
C1?	Bashkir	223T-298C-325C-327T-362C	[2]
C1?	Icelander	223T-298C-311C-325C-327T	[3]
C1?	Icelander	223T-298C-311C-325C-327T	[3]
C1?	Icelander	223T-298C-311C-325C-327T	[4]
C1?	Thai	223T-298C-311C-325C-327T-357C	[1]
C1?	Indian	093C-223T-298C-325C-327T-362C	[5]
C1?	Indian	093C-223T-298C-325C-327T-362C	[5]
C1?	Indian	093C-223T-298C-325C-327T-362C	[5]
C1?	Indian	093C-223T-298C-325C-327T-362C	[5]
C1?	Indian	093C-223T-298C-325C-327T-362C	[5]
C1?	Indian	093C-223T-298C-325C-327T-362C	[5]
C1?	German	223T-298C-311C-325C-327T	[6]
C1?	Canary Islander	223T-298C-325C-327T	[7]
C1a	Altaian	223T-298C-325C-327T-356C	[1]
C1a	Altaian	223T-298C-325C-327T-356C	[1]
C1a	Orok	223T-298C-325C-327T-356C	[8]
C1a	Orok	223T-298C-325C-327T-356C	[8]
C1a	Orok	223T-298C-325C-327T-356C	[8]
C1a	Orok	223T-298C-325C-327T-356C	[8]
C1a	Orok	223T-298C-325C-327T-356C	[8]
C1a	Orok	223T-298C-325C-327T-356C	[8]
C1a	Orok	223T-298C-325C-327T-356C	[8]
C1a	Highland Kirghiz	223T-298C-311C-325C-327T-356C	[9]
C1a	Japanese	223T-298C-325C-327T-356C	[10]
C1a	Japanese	223T-298C-325C-327T-356C	[11]
C1a	Japanese	093C-223T-298C-325C-327T-356C	[11]
C1a	Mongolian	093C-223T-298C-325C-327T-356C	[12]
C1a	Mongolian	093C-223T-298C-325C-327T-356C	[12]
C1a	Daur	093C-223T-298C-325C-327T-356C	[13]
C1a	Korean	093C-223T-298C-325C-327T-356C	[14]
C1a	Kalmyk	223T-298C-325C-327T-356C	[15]
C1a	Kalmyk	223T-298C-325C-327T-356C	[15]
C1a	Buryat	223T-298C-325C-327T-356C	[1]
C1a	Buryat	223T-298C-325C-327T-356C	[1]
C1a	Buryat	223T-298C-325C-327T-356C	[1]
C1a	Buryat	223T-298C-325C-327T-356C	[1]
C1a	Buryat	223T-298C-325C-327T-356C	[1]
C1a	Buryat	223T-298C-325C-327T-356C	[1]
C1a	Ulchi	223T-298C-325C-327T-356C	[16]
C1a	Ulchi	223T-298C-325C-327T-356C	[16]
C1a	Japanese	093C-223T-298C-325C-327T-356C	[17]
C1a	Buryat	223T-298C-325C-327T-356C	[17]
C1a	Japanese	093C-192T-223T-298C-325C-327T-356C	[18]

References

1. Balanovsky, personnal coommunication

2. Bermisheva M, Tambets K, Villems R, Khusnutdinova E (2002) Diversity of mitochondrial DNA haplotypes in ethnic populations of the Volga-Ural region of Russia. Mol Biol 36: 990-1001.

3. Helgason A, Sigureðardóttir S, Gulcher JR, Ward R, Stefánsson K (2000) mtDNA and the origin of the Icelanders: deciphering signals of recent population history. Am J Hum Genet 66:999-1016.

4. Helgason A, Nicholson G, Stefánsson K, Donnelly P (2003) A reassessment of genetic diversity in Icelanders: strong evidence from multiple loci for relative homogeneity caused by genetic drift. Ann Hum Genet 67:281-297.

5. Metspalu M, Kivisild T, Metspalu E, Parik J, Hudjashov G, et al. (2004) Most of the extant mtDNA boundaries in south and southwest Asia were likely shaped during the initial settlement of Eurasia by anatomically modern humans. BMC Genet 5:26.

6. Pfeiffer H, Brinkmann B, Hühne J, Rolf B, Morris A A, et al. (1999) Expanding the forensic German mitochondrial DNA control region database: genetic diversity as a function of sample size and microgeography. Int J Legal Med 112:291-298.

7. Rando JC, Cabrera VM, Larruga JM, Hernandez M, Gonzalez AM, et al. (1999). Phylogeographic patterns of mtDNA reflecting the colonization of the Canary Islands. Ann Hum Genet 63:413-428.

8. Bermisheva MA, Kutuev IA, Spitsyn VA, Villems R, Batyrova AZ, et al. (2005) Analysis of mitochondrial DNA variation in the population of Oroks. Genetika 41:78-84.

9. Comas D, Calafell F, Mateu E, Pérez-Lezaun A, Bosch E, et al. (1998) Trading genes along the silk road: mtDNA sequences and the origin of Central Asian populations. Am J Hum Genet 63:1824-1838.

10. Horai S, Hayasaka K (1990) Intraspecific nucleotide sequence differences in the major noncoding region of human mitochondrial DNA. Am J Hum Genet 46:828-42.

11. Imaizumi K, Parsons TJ, Yoshino M, Holland MM (2002) A new database of mitochondrial DNA hypervariable regions I and II sequences from 162 Japanese individuals. Int J Legal Med 116:68-73.

12. Kolman CJ, Sambuughin N, Bermingham E (1996) Mitochondrial DNA analysis of Mongolian populations and implications for the origin of New World founders. Genetics 142:1321-34.

13. Kong Q P, Yao Y G, Sun C, Bandelt H J, Zhu C L, Zhang Y P (2003) Phylogeny of East Asian mitochondrial DNA lineages inferred from complete sequences. Am J Hum Genet 73: 671-676.

14. Lee HY, Yoo JE, Park MJ, Chung U, Shin KJ (2006) Mitochondrial DNA control region sequences in Koreans: identification of useful variable sites and phylogenetic analysis for mtDNA data quality control. Int J Legal Med 120:5-14.

15. Nasidze I, Quinque D, Dupanloup I, Cordaux R, Kokshunova L, et al. (2005) Genetic evidence for the Mongolian ancestry of Kalmyks. Am J Phys Anthropol 128:846-54.

16. Starikovskaya EB, Sukernik RI, Derbeneva OA, Volodko NV, Ruiz-Pesini E, et al. (2005) Mitochondrial DNA diversity in indigenous populations of the southern extent of Siberia, and the origins of Native American haplogroups. Ann Hum Genet 69:67-89.

17. Tajima A, Hayami M, Tokunaga K, Juji T, Matsuo M, et al. (2004) Genetic origins of the Ainu inferred from combined DNA analyses of maternal and paternal lineages. J Hum Genet 49:187-93.

18. Tanaka M, Cabrera VM, González AM, Larruga JM, Takeyasu T, et al. (2004) Mitochondrial genome variation in eastern Asia and the peopling of Japan. Genome Res 14:1832-50.
