# Supplementary material for: Ancient DNA Reveals Prehistoric Gene-Flow from Siberia in the Complex Human Population History of North East Europe
Source: PLoS Genet. 2013 Feb 14;9(2):e1003296. doi: 10.1371/journal.pgen.1003296 (PMC3573127; doi:10.1371/journal.pgen.1003296)
Supplement: Table S2 — Grave and museum collection number for Yuzhnyy Oleni Ostrov, Popovo and Bol'shoy Oleni Ostrov specimens. (PDF) [file pgen.1003296.s005.pdf]

Table S2. Grave and museum collection number for Yuzhnyy Oleni Ostrov, Popovo and Bol’shoy Oleni Ostrov specimens.

Specimens	MAE RAS collection number	Grave number
**Yuzhnyy Oleni Ostrov**		
UzOO 7	5773-7	56
UzOO 8	5773-8	57
UzOO 16	5773-16	60
UzOO 40	5773-40	39/1
UzOO 43	5773-43	95
UzOO 46	5773-46	69
UzOO 70	5773-70	118
UzOO 74	5773-74	142
UzOO 77	5773-77	54
**Popovo**		
Po2	6836-2	2
Po4	6836-4	4
**Bol'shoy Oleni Ostrov**		
BOO49-1	4952-1	2
BOO49-2	4952-2	3
BOO49-3	4952-3	4
BOO49-4	4952-4	5
BOO49-5	4952-5	
BOO49-6	4952-6	8
BOO57-1	5715-1	III
BOO57-3	5715-3	V
BOO72-1	7265-1	12, s.1
BOO72-2	7265-2	14, s.1
BOO72-3	7265-3	15, s.1
BOO72-4	7265-4	16, s.1
BOO72-5	7265-5	16, s.2
BOO72-6	7265-6	16, s.3
BOO72-7	7265-7	16, s.4
BOO72-8	7265-8	17, s.1
BOO72-9	7265-9	17, s.2
BOO72-10	7265-10	17, s.3
BOO72-11	7265-11	17, s.4
BOO72-12	7265-12	19, s.1
BOO72-13	7265-13	19, s.2
BOO72-14	7265-14	19, s.3
BOO72-15	7265-15	19, s.4

MAE RAS, Museum of Anthropology and Enthnography, Russian Academy of Sciences;
s, sepulture
