# Supplementary material for: Ancient DNA Reveals Prehistoric Gene-Flow from Siberia in the Complex Human Population History of North East Europe
Source: PLoS Genet. 2013 Feb 14;9(2):e1003296. doi: 10.1371/journal.pgen.1003296 (PMC3573127; doi:10.1371/journal.pgen.1003296)
Supplement: Table S3 — Results of SNP typing in the mtDNA coding region using the GenoCore22 SNaPshot assay. SNPs typed on the L-strand are reported in capital letters in the reference rCRS profile, whereas SNPs typed on the H-strand are reported in small letters. Missing data signifies allelic dropout or fluorescence signal below the background threshold (100 relative fluorescent units, rfu). ‘g/a’ indicates the presence of a mixed signal for the position interrogated. A mixed signal was repeatedly obtained at position 8994 (haplogroup W) with the detection of an additional G base. However, the rest of the profile never could support phylogenetically the presence of the G base at this particular position. For each individual, profiles were obtained from two independent extracts, except for individual BOO72-9 for which a second samples was not available and for UZOO-77, BOO57-1, BOO72-10, BOO72-4, BOO72-7, BOO72-15, and BOO72-1, for which the second individual was extracted in an independent laboratory. rCRS, revised Cambridge Reference Sequence; hg, haplogroup. (PDF) [file pgen.1003296.s006.pdf]

Table S3. Results of SNP typing in the mtDNA coding region using the GenoCore22 SNaPshot assay.

		13928_R9	3594_L3'4	10550_K	11467_U	4248_A	8994_W	13263_C	13368_T	11719_preHV, R0	8280delB	4580_V	6371_X	10238_N1	7028_H	10034_I	5178_D	14766_HV	12705_R	10873_N	12612_J	2758_L2'6	10400_M	hg
**rCRS**		c	G	t	A	T	G	t	G	c	A	c	g	T	g	T	g	G	g	a	a	G	g	**H**
**Sample**	**Extract #**																							
**aUz**																								
UZOO-43	1	C	G	T	**G**	T	G	T	G	T	A	C	G	T	A	T	G	A	G	A	A	G	G	**U**
	2	C	G	T	**G**	T	G	T	G	T	A	C	G	T	A	T	G	A	G	A	A	G	G	**U**
UZOO-46	1	C	G	T	**G**	T	G	T	G	T	A	C	G	T	A	T	G	A	G	A	A	G	G	**U**
	2	C	G	T	**G**	T	G	T	G	T	A	C	G	T	g/a	T	G	A	G		A	G	G	**U**
UZOO-16	1	C	G	T	**G**	T	G	T	G	T	A	C	G	T	A	T	G	A	G	A	A	G	G	**U**
	2	C	G	T	**G**	T	G	T	G	T	A	C	G	T	g/a	T	G	A	G	A	A	G	G	**U**
UZOO-40	1	C	G	T	**G**	T	G	T	G	T	A	C	G	T	A	T	G	A	G	A	A	G	G	**U**
	2	C	G	T	**G**	T	G	T	G	T	A	C	G	T	A	T	G	A	G	A	A	G	G	**U**
UZOO-70	1	C	G	T	**G**	T	G	T	G	T	A	C	G	T	A	T	G	A	G	A	A	G	G	**U**
	2	C	G	T	**G**	T	g/a	T	G	T	A	C	G	T	A	T	G	A	G	A	A	G	G	**U**
UZOO-77	1	C	G	T	A	T	G	T	G	**C**	A	C	G	T	**G**	T	G	**G**	G	A	A	G	G	**H**
UZOO-7	1	C		T	A	T	G	**C**	G	T	A	C	G	T	A	T	G			G	A	G	A	**C**
	2	C	G	T	A	T	g/a	**C**	G	T	A	C	G	T	A	T	G		A	G	A	G	A	**C**
UZOO-8	1	C	G	T	A	T	G	**C**	G	T	A	C	G	T	A	T	G	A	A	G	A	G	A	**C**
	2	C	G	T		T	g/a	**C**	G	T	A		G	T	g/a	T	G			G	A	G		**C**
UZOO-74	1	C	G	T	A	T	G	**C**	G	T	A	C	G	T	A	T	G		A	G	A	G	A	**C**
	2	C	G	T	A	T	G	**C**	G	T	A	C	G	T	A	T	G		A	G	A	G	A	**C**

SNPs typed on the L-strand are reported in capital letters in the reference rCRS profile, whereas SNPs typed on the H-strand are reported in small letters. Missing data signifies allelic dropout or fluorescence signal below the background threshold (100 relative fluorescent units, rfu). 'g/a' indicates the presence of a mixed signal for the position interrogated. A mixed signal was repeatedly obtained at position 8994 (haplogroup W) with the detection of an additional G base. However, the rest of the profile never could support phylogenetically the presence of the G base at this particular position. For each individual, profiles were obtained from two independent extracts, except for individual BOO72-9 for which a second samples was not available and for UZOO-77, BOO57-1, BOO72-10, BOO72-4, BOO72-7, BOO72-15, and BOO72-1, for which the second individual was extracted in an independent laboratory.
rCRS, revised Cambridge Reference Sequence; hg, haplogroup.

		13928_R9	3594_L3'4	10550_K	11467_U	4248_A	8994_W	13263_C	13368_T	11719_preHV, R0	8280delB	4580_V	6371_X	10238_N1	7028_H	10034_I	5178_D	14766_HV	12705_R	10873_N	12612_J	2758_L2'6	10400_M	hg
**aPo**																								
Po4	1	C	G	T	**G**	T	g/a		G	T	A		G	T	A	T	G		G		A	G		**U**
	2	C	G	T	**G**	T	g/a	T	G	T	A		G	T	A	T	G				A	G	G	**U**
Po2	1	C	G	T	**G**	T	G	T	G	T	A	C	G	T	A	T	G	A	G	A	A	G	G	**U**
	2	C	G	T	**G**	T	g/a	T	G	T	A		G	T	A	T	G		G	A	A	G	G	**U**
**aBOO**																								
BOO49-3	1	C	G	T	**G**	T	G	T	G	T	A	C	G	T	A	T	G	A	G	A	A	G	G	**U**
	2	C	G	T	**G**	T	G	T	G	T	A	C	G	T	A	T	G	A	G	A	A	G	G	**U**
BOO57-1	1	C	G	T	**G**	T	G	T	G	T	A	C	G	T	A	T	G	A	G	A	A	G	G	**U**
BOO49-1	1	C	G	T	**G**	T	G	T	G	T	A	C	G	T	A	T	G	A	G	A	A	G	G	**U**
	2	C	G	T	**G**	T	G	T	G	T	A	C	G	T	A	T	G	A	G	A	A	G	G	**U**
BOO72-11	1	C	G	T	**G**	T	G	T	G	T	A	C	G	T	A	T	G	A	G	A	A	G	G	**U**
	2	C	G	T	**G**	T	G	T	G	T	A	C	G	T	A	T	G	A	G	A	A	G	G	**U**
BOO72-9	1	C	G	T	**G**	T	G	T	G	T	A	C	G	T	A	T	G	A	G	A	A	G	G	**U**
BOO72-10	1	C	G	T	**G**	T	G	T	G	T	A	C	G	T	A	T	G	A	G	A	A	G	G	**U**
BOO72-14	1	C	G	T	**G**	T	G	T	G	T	A	C	G	T	A	T	G	A	G	A	A	G	G	**U**
	2	C	G	T	**G**	T	G	T	G	T	A	C	G	T	A	T	G	A	G	A	A	G	G	**U**
BOO72-8	1	C	G	T	**G**	T	G	T	G	T	A	C	G	T	A	T	G	A	G	A	A	G	G	**U**
	2	C	G	T	**G**	T	G	T	G	T	A	C	G	T	A	T	G	A	G	A	A	G	G	**U**
BOO72-4	1	C	G	T	A	T	G	T	**A**	T	A	C	G	T	A	T	G	A	G	A	A	G	G	**T**
BOO49-2	1	C	G	T	A	T	G	**C**	G	T	A		G	T	A	T	G			G	A	G		**C**
	2	C	G	T	A	T	G	**C**	G	T	A	C	G	T	A	T	G			G		G		**C**
BOO49-4	1	C	G	T	A	T	G	**C**	G	T	A	C	G	T	A	T	G	A	A	G	A	G	A	**C**
	2	C	G	T	A	T	G	**C**	G	T	A		G	T	A	T	G			G		G	A	**C**
BOO57-3	1	C	G	T	A	T	G	**C**	G	T	A	C	G	T	A	T	G		A	G	A	G	A	**C**
	2	C	G	T	A	T	G	**C**	G	T	A	C	G	T	A	T	G	A	A	G	A	G	A	**C**
BOO72-2	1	C	G	T	A	T	G	**C**	G	T	A	C	G	T	A	T	G	A	A	G	A	G	A	**C**
	2	C	G	T	A	T	G	**C**	G	T	A	C	G	T	A	T	G	A	A	G	A	G	A	**C**
BOO72-7	1	C	G	T	A	T	G	**C**	G	T	A		G		A	T	G			G	A	G	A	**C**
BOO72-12	1	C	G	T	A	T	G	**C**	G	T	A	C	G	T	A	T	G		A	G	A	G	A	**C**
	2	C	G	T	A	T	G	**C**	G	T	A		G	T	A	T	G		A		A	G	A	**C**
BOO72-5	1	C	G	T	A	T	G	**C**	G	T	A	C	G	T	A	T	G		A	G	A	G	A	**C**
	2	C	G	T	A	T	G	**C**	G	T	A	C	G	T	A	T	G	A	A	G	A	G	A	**C**
BOO72-6	1	C	G	T	A	T	G	**C**	G	T	A	C	G	T	A	T	G	A	A	G	A	G	A	**C**
	2	C	G	T	A	T	G	**C**	G	T	A	C	G	T	A	T	G		A	G	A	G	A	**C**
BOO49-6	1	C	G	T	A	T	G	T	G	T	A	C	G	T	A	T	**T**	A	A	G	A	G	A	**D**
	2		G	T	A	T	G	T	G	T	A	C	G	T	A	T	**T**	A		G	A	G		**D**
BOO72-13	1	C	G	T	A	T	G	T	G	T	A	C	G	T	A	T	**T**	A	A	G	A	G	A	**D**
	2	C	G	T	A	T	G	T	G	T	A	C	G	T	A	T	**T**	A	A	G	A	G	A	**D**
BOO72-15	1	C	G	T	A	T	G	T	G	T	A	C	G	T	A	T	**T**	A	A	G	A	G	A	**D**
BOO49-5	1		G	T	A	T	G	T	G	T	A	C	G	T	A	T	G		A	G	A	G	A	**M**
	2	C	G	T	A	T	G	T	G	T	A		G	T	A	T	G	A	A	G	A	G	A	**M**
BOO72-3	1	C	G	T	A	T	G	T	G	T	A	C	G	T	A	T	G			G	A	G	A	**M**
	2	C	G	T	A	T	G	T	G	T	A	C	G	T	A	T	G	A	A	G	A	G	A	**M**
BOO72-1	1	C	G	T	A	T	G	T	G	T	A		G	T	A	T	G			G	A	G	A	**M**

SNPs typed on the L-strand are reported in capital letters in the reference rCRS profile, whereas SNPs typed on the H-strand are reported in small letters. Missing data signifies allelic dropout or fluorescence signal below the background threshold (100 relative fluorescent units, rfu). 'g/a' indicates the presence of a mixed signal for the position interrogated. A mixed signal was repeatedly obtained at position 8994 (haplogroup W) with the detection of an additional G base. However, the rest of the profile never could support phylogenetically the presence of the G base at this particular position. For each individual, profiles were obtained from two independent extracts, except for individual BOO72-9 for which a second samples was not available and for UZOO-77, BOO57-1, BOO72-10, BOO72-4, BOO72-7, BOO72-15, and BOO72-1, for which the second individual was extracted in an independent laboratory.

rCRS, revised Cambridge Reference Sequence; hg, haplogroup.
