# Supplementary material for: Ancient DNA Reveals Prehistoric Gene-Flow from Siberia in the Complex Human Population History of North East Europe
Source: PLoS Genet. 2013 Feb 14;9(2):e1003296. doi: 10.1371/journal.pgen.1003296 (PMC3573127; doi:10.1371/journal.pgen.1003296)
Supplement: Table S4 — Results of quantitative PCR. (PDF) [file pgen.1003296.s007.pdf]

Table S4. Results of quantitative PCR.

PCR:	L16209/H16303					
**Site**	**Sample**	**Cycle Threshold (Ct)**	**Average Ct**	**Stdev**	**Stdev/AverageCt %**	**Copies/uL**
aUz	UZOO43	38.87	38.94	0.08	0.20	1 158
aUz	UZOO43	39.02				
aUz	UZOO43	38.92				
aUz	UZOO74	42.32	42.03	0.51	1.22	160
aUz	UZOO74	41.44				
aUz	UZOO74	42.33				
aBOO	BOO72-1	33.88	33.95	0.18	0.53	28 117
aBOO	BOO72-1	34.16				
aBOO	BOO72-1	33.82				
aBOO	BOO79-9	37.38	36.65	2.12	5.79	5 004
aBOO	BOO79-9	38.31				
aBOO	BOO79-9	34.26				
Site4	sample1	33.70	34.05	0.46	1.35	26 430
Site4	sample1	33.88				
Site4	sample1	34.57				
Site4	sample2	33.79	33.51	0.25	0.74	37 424
Site4	sample2	33.34				
Site4	sample2	33.39				
Site4	sample3	34.43	34.56	0.51	1.47	19 069
Site4	sample3	35.12				
Site4	sample3	34.13				
**PCR:**	**L16209/H16348**					
**Site**	**Sample**	**Cycle Threshold (Ct)**	**Average Ct**	**Stdev**	**Stdev/AverageCt %**	**Copies/uL**
aUz	UZOO43	34.54	34.69	0.15	0.43	6
aUz	UZOO43	34.68				
aUz	UZOO43	34.84				
aUz	UZOO74	35.30	35.03	0.24	0.68	5
aUz	UZOO74	34.84				
aUz	UZOO74	34.96				
aBOO	BOO72-1	31.11	30.89	0.64	2.06	70
aBOO	BOO72-1	30.17				
aBOO	BOO72-1	31.38				
aBOO	BOO79-9	30.42	30.44	0.05	0.17	88
aBOO	BOO79-9	30.50				
aBOO	BOO79-9	30.40				
Site4	sample1	31.76	32.11	0.62	1.92	31
Site4	sample1	31.74				
Site4	sample1	32.82				
Site4	sample2	30.05	30.20	0.14	0.47	103
Site4	sample2	30.33				
Site4	sample2	30.21				
Site4	sample3	31.86	31.76	0.57	1.78	39
Site4	sample3	31.15				
Site4	sample3	32.27				
