# Supplementary material for: Ancient DNA Reveals Prehistoric Gene-Flow from Siberia in the Complex Human Population History of North East Europe
Source: PLoS Genet. 2013 Feb 14;9(2):e1003296. doi: 10.1371/journal.pgen.1003296 (PMC3573127; doi:10.1371/journal.pgen.1003296)
Supplement: Table S5 — Details of ancient and modern-day populations used in comparative analyses. (PDF) [file pgen.1003296.s008.pdf]

Table S5. Details of ancient and modern-day populations used in comparative analyses.

MODERN-DAY POPULATIONS				
**Population names**	**label**	**N**	**References**	**BayeSSC pools**
Albanians	ALB	281	[1,2,3]	
Aleuts	ale	199	[4]	
Kizhi	alt	90	[5]	
Armenians	ARM	192	[6]	
Aromuns	aro	133	[2]	
Austrians	AUT	117	[7,8]	
Azeris	AZE	88	[9,10]	
Bashkirs	BA	207	[11]	
Byelorussians	BEL	352	[3,12]	
Bulgarians	BGR	141	[9,13]	
Bosnians	BIH	322	[14,15]	
Buryats	BU	411	[5,16,17]	
Swiss	CHE	230	[18,19]	
Chukchi	CHU	262	[4,17,20]	
Chuvash	CU	92	[9,11]	
Cyprus	CYP	91	[21]	
Czech	CZE	449	[3,9,22,23]	CE
Germans	DEU	1406	[24,25,26,27,28]	CE
Eskimo	esk	825	[4,20,29,30,31]	
Spanish	ESP	704	[32,33,34,35,36,37]	
Estonians	EST	662	[3,18,39,39,40]	NEE
Evenks	eve	407	[5,16,17,41,42]	
Finns	FIN	508	[43,44,45]	NEE
French	FRA	998	[46,47]	
British	GBR	2418	[9,24,48,49,50]	
Georgians	GEO	158	[3,10]	
Greeks	GRC	656	[2,3,9,21]	
Croatians	HRV	980	[3,51]	
Hungarians	HUN	190	[3,52]	
Irish	IRL	300	[52,53]	
Iranians	IRN	517	[10,54]	
Iraq	IRQ	168	[9,55]	
Iraqi	ISL	448	[24,38,56]	
Sardinians	IT-88	115	[9]	
Italians	ITA	936	[9,57,58,59,60,61,62]	
Jordanians	JOR	146	[63]	
Kabardians	kab	163	[3,9]	
Kets	ket	104	[3,64]	
Khamnigans	kham	99	[17]	
Khants	khan	318	[3,65]	

Khakassians	KK	110	[5,16,17]	
Komi	KO	127	[11]	
Koryaks	kor	147	[66]	
Karelians	KR	305	[40]	NEE
Kurds	kur	73	[9,10]	
Lithuanians	LTU	180	[3]	NEE
Latvians	LVA	413	[40,67]	NEE
Mansi	man	161	[64,65]	
Mari	ME	136	[11]	
Mongolians	MNG	262	[17,68,69,70]	
Mordvinians	MO	99	[11]	
Nenets Asian	NEN_A	79	[3]	
Nenets European	NEN_E	128	[29,71]	
Nganasans	nga	118	[4,64,72]	
Nivkhs	niv	113	[5,41]	
Nogays	nog	206	[73]	
Norwegians	NOR	663	[9,49,74,75,76]	
Poles	POL	583	[3,9,77]	CE
Portuguese	PRT	848	[37,78]	
Palestinians	PSE	117	[9]	
Romanians	ROU	197	[2,9]	
Yakuts	SA	770	[3,16,17,79,80,81]	
Saami	saa	559	[38,44,71,74,82,83]	saa
Saudi Arabians	SAU	325	[3,84]	
Ossets	SE	289	[3,9]	
Selkups	sel	120	[3]	
Shors	sho	82	[17]	
Slovaks	SVK	510	[85]	
Slovenians	SVN	233	[77,86]	
Swedes	SWE	646	[3]	
Syrians	SYR	169	[3,9]	
Tatars	TA	225	[11]	
Telenghits	tel	71	[17]	
Tofalars	tof	104	[5,16]	
Tubalars	tub	72	[17]	
Turks	TUR	608	[3,9,10,13]	
Tuvinians	tuv	645	[3,5,16,17,42,71]	
Udmurts	UD	109	[11]	
Ukrainians	UKR	610	[3]	
Ulchi	ulc	166	[5]	
Yukagir	yuk	153	[4,42]	

ANCIENT POPULATIONS				
**Population name**	**label**	**N**	**References**	**Dates**
Bol'shoy Oleni Ostrov individuals	aBOO	23	this study	3,500 uncal. yBP
Bol'shoy Oleni Ostrov individuals (non-redundant haplotypes)	aBOO2	9	this study	3,500 uncal. yBP
Confederated nomads of the Xiongnu	aEG	46	[87]	2,200-2,300 yBP
Central/East Europe hunter-gatherers	aHG	22	[88,89]	4,250-30,000 yBP
Nomads from Kazakhstan	aKAZ	25	[90]	2,100-3,400 yBP
Siberian Kurgans	aKUR	26	[91]	1,600-3,800 yBP
Central Germany Linear Pottery Culture Neolithic individuals	aLBK	42	[92]	7,000-7,500 yBP
Lokomotiv Kitoi Neolithic individuals	aLOK	30	[93]	6,130-7,140 yBP
Popovo individuals	aPo	2	this study	7,000 uncal. yBP
Scandinavian Pitted-Ware Culture foragers	aPWC	19	[94]	4,500-5,300 yBP
Spanish Neolithic individuals	aSP	11	[95]	5,000-5,500 yBP
Ust'Ida Neolithic population	aUST	17	[96]	4,000-5,800 yBP
Yuzhnyy Oleni Ostrov individuals	aUz	9	this study	7,500 yBP
Uzhnyi Oleni Ostrov/Popovo individuals	aUzPo	11	this study	7,500 uncal. yBP
Uzhnyi Oleni Ostrov/Popovo individuals (non-redundant haplotypes)	aUzPo2	7	this study	7,500 uncal. yBP

BayeSSC, Bayesian SerialSimCoal; CEE, central Europeans ; NEE, North East Europeans; saa, Saami.

References

1. Belledi M, Poloni ES, Casalotti R, Conterio F, Mikerezi I, et al. (2000) Maternal and paternal lineages in Albania and the genetic structure of Indo-European populations. Eur J Hum Genet 8: 480-486.
2. Bosch E, Calafell F, Gonzalez-Neira A, Flaiz C, Mateu E, et al. (2006) Paternal and maternal lineages in the Balkans show a homogeneous landscape over linguistic barriers, except for the isolated Aromuns. Ann Hum Genet 70: 459-487.
3. Balanovsky, personal communication
4. Volodko NV, Starikovskaya EB, Mazunin IO, Eltsov NP, Naidenko PV et al. (2008) Mitochondrial genome diversity in arctic Siberians, with particular reference to the evolutionary history of Beringia and Pleistocenic peopling of the Americas. Am J Hum Genet 82:1084-100.
5. Starikovskaya EB, Sukernik RI, Derbeneva OA, Volodko NV, Ruiz-Pesini E, et al. (2005) Mitochondrial DNA diversity in indigenous populations of the southern extent of Siberia, and the origins of Native American haplogroups. Ann Hum Genet 69: 67-89.
6. Metspalu et al., unpublished.

7. Handt O, Richards M, Trommsdorff M, Kilger C, Simanainen J, et al. (1994) Molecular genetic analyses of the Tyrolean Ice Man. Science 264: 1775-1778.

8. Parson W, Parsons TJ, Scheithauer R, Holland MM (1998) Population data for 101 Austrian Caucasian mitochondrial DNA d-loop sequences: application of mtDNA sequence analysis to a forensic case. Int J Legal Med 111: 124-132.

9. Richards M, Macaulay V, Hickey E, Vega E, Sykes B, et al. (2000) Tracing European founder lineages in the Near Eastern mtDNA pool. Am J Hum Genet 67: 1251-1276.

10. Quintana-Murci L, Chaix R, Wells RS, Behar DM, Sayar H, et al. (2004) Where west meets east: the complex mtDNA landscape of the southwest and Central Asian corridor. Am J Hum Genet 74: 827-845.

11. Bermisheva M, Tambets K, Villems R, Khusnutdinova E (2002) [Diversity of mitochondrial DNA haplotypes in ethnic populations of the Volga-Ural region of Russia]. Mol Biol (Mosk) 36: 990-1001.

12. Belyaeva O, Bermisheva M, Khrunin A, Slominsky P, Bebyakova N, et al. (2003) Mitochondrial DNA Variations in Russian and Belorussian. Hum Biol 75: 647-660.

13. Calafell F, Underhill P, Tolun A, Angelicheva D, Kalaydjieva L (1996) From Asia to Europe: mitochondrial DNA sequence variability in Bulgarians and Turks. Ann Hum Genet 60 (Pt 1): 35-49.

14. Malyarchuk BA, Grzybowski T, Derenko MV, Czarny J, Drobnic K, et al. (2003) Mitochondrial DNA variability in Bosnians and Slovenians. Ann Hum Genet 67:412-25.

15. Harvey, Unpublished.

16. Derenko MV, Maliarchuk BA, Zakharov IA (2002) Origin of caucasoid-specific mitochondrial DNA lineages in the ethnic populations of the Altai-Sayan region. Genetika 38:1292-7.

17. Derenko M, Malyarchuk B, Grzybowski T, Denisova G, Dambueva I (2007) Phylogeographic analysis of mitochondrial DNA in northern Asian populations. Am J Hum Genet 81:1025-41.

18. Pult I, Sajantila A, Simanainen J, Georgiev O, Schaffner W, et al. (1994) Mitochondrial DNA sequences from Switzerland reveal striking homogeneity of European populations. Biol Chem Hoppe Seyler 375: 837-840.

19. Dimo-Simonin N, Grange F, Taroni F, Brandt-Casadevall C, Mangin P (2000) Forensic evaluation of mtDNA in a population from south west Switzerland. Int J Legal Med 113: 89-97.

20. Starikovskaya YB, Sukernik RI, Schurr TG, Kogelnik AM, Wallace DC (1998) mtDNA diversity in Chukchi and Siberian Eskimos: implications for the genetic history of Ancient Beringia and the peopling of the New World. Am J Hum Genet 63:1473-91.

21. Irwin J, Saunier J, Strouss K, Paintner C, Diegoli T, et al. (2008) Mitochondrial control region sequences from northern Greece and Greek Cypriots. Int J Legal Med 122: 87-89.

22. Vanecek T, Vorel F, Sip M (2004) Mitochondrial DNA D-loop hypervariable regions: Czech population data. Int J Legal Med 118: 14-18.

23. Malyarchuk BA, Vanecek T, Perkova MA, Derenko MV, Sip M (2006) Mitochondrial DNA variability in the Czech population, with application to the ethnic history of Slavs. Hum Biol 78: 681-696.

24. Richards M, Corte-Real H, Forster P, Macaulay V, Wilkinson-Herbots H, et al. (1996) Paleolithic and neolithic lineages in the European mitochondrial gene pool. Am J Hum Genet 59: 185-203.

25. Baasner A, Schafer C, Junge A, Madea B (1998) Polymorphic sites in human mitochondrial DNA control region sequences: population data and maternal inheritance. Forensic Sci Int 98: 169-178.

26. Lutz S, Weisser HJ, Heizmann J, Pollak S (1998) Location and frequency of polymorphic positions in the mtDNA control region of individuals from Germany. Int J Legal Med 111: 67-77.

27. Pfeiffer H, Brinkmann B, Huhne J, Rolf B, Morris AA, et al. (1999) Expanding the forensic German mitochondrial DNA control region database: genetic diversity as a function of sample size and microgeography. Int J Legal Med 112: 291-298..

28. Poetsch M, Wittig H, Krause D, Lignitz E (2003) Mitochondrial diversity of a northeast German population sample. Forensic Sci Int 137: 125-132.

29. Saillard J, Forster P, Lynnerup N, Bandelt HJ, Nørby S (2000) mtDNA variation among Greenland Eskimos: the edge of the Beringian expansion. Am J Hum Genet 67:718-26.

30. Helgason A, Pálsson G, Pedersen HS, Angulalik E, Gunnarsdóttir ED, et al. (2006) mtDNA variation in Inuit populations of Greenland and Canada: migration history and population structure. Am J Phys Anthropol 130:123-34.

31. Simonson et al., unpublished.

32. Corte-Real HB, Macaulay VA, Richards MB, Hariti G, Issad MS, et al. (1996) Genetic diversity in the Iberian Peninsula determined from mitochondrial sequence analysis. Ann Hum Genet 60: 331-350.

33. Salas A, Comas D, Lareu MV, Bertranpetit J, Carracedo A (1998) mtDNA analysis of the Galician population: a genetic edge of European variation. Eur J Hum Genet 6: 365-375.

34. Crespillo M, Luque JA, Paredes M, Fernandez R, Ramirez E, et al. (2000) Mitochondrial DNA sequences for 118 individuals from northeastern Spain. Int J Legal Med 114: 130-132.

35. Larruga JM, Diez F, Pinto FM, Flores C, Gonzalez AM (2001) Mitochondrial DNA characterisation of European isolates: the Maragatos from Spain. Eur J Hum Genet 9: 708-716.

36. Maca-Meyer N, Sanchez-Velasco P, Flores C, Larruga JM, Gonzalez AM, et al. (2003) Y chromosome and mitochondrial DNA characterization of Pasiegos, a human isolate from Cantabria (Spain). Ann Hum Genet 67: 329-339.

37. Gonzalez AM, Karadsheh N, Maca-Meyer N, Flores C, Cabrera VM, et al. (2008) Mitochondrial DNA variation in Jordanians and their genetic relationship to other Middle East populations. Ann Hum Biol 35: 212-231.

38. Sajantila A, Lahermo P, Anttinen T, Lukka M, Sistonen P, et al. (1995) Genes and languages in Europe: an analysis of mitochondrial lineages. Genome Res 5: 42-52.

39. Sajantila A, Salem AH, Savolainen P, Bauer K, Gierig C, et al. (1996) Paternal and maternal DNA lineages reveal a bottleneck in the founding of the Finnish population. Proc Natl Acad Sci U S A 93:12035-9.

40. Lappalainen T, Laitinen V, Salmela E, Andersen P, Huoponen K (2008) Migration waves to the Baltic Sea region. Ann Hum Genet 72, 337-348.
41. Tajima A, Hayami M, Tokunaga K, Juji T, Matsuo M, et al. (2004) Genetic origins of the Ainu inferred from combined DNA analyses of maternal and paternal lineages. J Hum Genet 49:187-93.
42. Pakendorf B, Novgorodov IN, Osakovskij VL, Danilova AP, Protod'jakonov AP, et al. (2006) Investigating the effects of prehistoric migrations in Siberia: genetic variation and the origins of Yakuts. Hum Genet 120: 33.
43. Lahermo P, Sajantila A, Sistonen P, Lukka M, Aula P, et al. (1996) The genetic relationship between the Finns and the Finnish Saami (Lapps): analysis of nuclear DNA and mtDNA. Am J Hum Genet 58: 1309-1322.
44. Kittles RA, Bergen AW, Urbanek M, Virkkunen M, Linnoila M, et al. (1999) Autosomal, mitochondrial, and Y chromosome DNA variation in Finland: evidence for a male-specific bottleneck. Am J Phys Anthropol 108: 381-399.
45. Meinila M, Finnila S, Majamaa K (2001) Evidence for mtDNA admixture between the Finns and the Saami. Hum Hered 52: 160-170.
46. Dubut V, Chollet L, Murail P, Cartault F, Beraud-Colomb E, et al. (2004) mtDNA polymorphisms in five French groups: importance of regional sampling. Eur J Hum Genet 12: 293-300.
47. Richard C, Pennarun E, Kivisild T, Tambets K, Tolk HV, et al. (2007) An mtDNA perspective of French genetic variation. Ann Hum Biol 34: 68-79.
48. Piercy R, Sullivan KM, Benson N, Gill P (1993) The application of mitochondrial DNA typing to the study of white Caucasian genetic identification. Int J Legal Med 106:85-90.
49. Helgason A, Hickey E, Goodacre S, Bosnes V, Stefansson K, et al. (2001) mtDNA and the Islands of the North Atlantic: Estimating the Proportions of Norse and Gaelic Ancestry. Am J Hum Genet 68: 723-737.
50. Sykes B (2006) Blood of the Isles: Exploring the Genetic Roots of Our Tribal History: Bantam. 306 p.

51. Babalini C, Martinez-Labarga C, Tolk HV, Kivisild T, Giampaolo R, et al. (2005) The population history of the Croatian linguistic minority of Molise (southern Italy): a maternal view. Eur J Hum Genet 13: 902-912.

52. Bogácsi-Szabó E, Kalmár T, Csányi B, Tömöry G, Czibula A, et al. (2005) Mitochondrial DNA of ancient Cumanians: culturally Asian steppe nomadic immigrants with substantially more western Eurasian mitochondrial DNA lineages. Hum Biol 77:639-62.

53. McEvoy B, Richards M, Forster P, Bradley DG (2004) The Longue Duree of genetic ancestry: multiple genetic marker systems and Celtic origins on the Atlantic facade of Europe. Am J Hum Genet 75: 693-702.

54. Metspalu M, Kivisild T, Metspalu E, Parik J, Hudjashov G, et al. (2004) Most of the extant mtDNA boundaries in South and Southwest Asia were likely shaped during the initial settlement of Eurasia by anatomically modern humans. BMC Genetics 5: 26. doi:10.1186/1471-2156-5-26

55. Al-Zahery N, Semino O, Benuzzi G, Magri C, Passarino G, et al. (2003) Y-chromosome and mtDNA polymorphisms in Iraq, a crossroad of the early human dispersal and of post-Neolithic migrations. Mol Phylogenet Evol 28: 458-472.

56. Helgason A, Sigurdardottir S, Gulcher JR, Stefansson K, Ward R, et al. (2000) Sampling Saturation and the European MtDNA Pool: Implications for Detecting Genetic Relationships among Populations. Archaeogenetics: DNA and the population prehistory of Europe. Cambridge: McDonald Institute for Archaeological Research. pp. 285-294.

57. Francalacci P, Bertranpetit J, Calafell F, Underhill PA (1996) Sequence diversity of the control region of mitochondrial DNA in Tuscany and its implications for the peopling of Europe. Am J Phys Anthropol 100: 443-460.

58. Cali F, Le Roux MG, D'Anna R, Flugy A, De Leo G, et al. (2001) MtDNA control region and RFLP data for Sicily and France. Int J Legal Med 114: 229-231.

59. Mogentale-Profizi N, Chollet L, Stevanovitch A, Dubut V, Poggi C, et al. (2001) Mitochondrial DNA sequence diversity in two groups of Italian Veneto speakers from Veneto. Ann Hum Genet 65: 153-166.

60. Tagliabracci A, Turchi C, Buscemi L, Sassaroli C (2001) Polymorphism of the mitochondrial DNA control region in Italians. Int J Legal Med 114: 224-228.

61. Achilli A, Olivieri A, Pala M, Metspalu E, Fornarino S, et al. (2007) Mitochondrial DNA variation of modern Tuscans supports the near eastern origin of Etruscans. Am J Hum Genet 80: 759-768.

62. Varesi et al., unpublished.

63. Cabrera et al., unpublished.

64. Derbeneva OA, Starikovskaia EB, Volod'ko NV, Wallace DC, Sukernik RI (2002) Mitochondrial DNA variation in Kets and Nganasans and the early peoples of Northern Eurasia. Genetika 38:1554-60.

65. Pimenoff VN, Comas D, Palo JU, Vershubsky G, Kozlov A, et al. (2008) Northwest Siberian Khanty and Mansi in the junction of West and East Eurasian gene pools as revealed by uniparental markers. Eur J Hum Genet 16:1254-64.

66. Schurr TG, Sukernik RI, Starikovskaya YB, Wallace DC (1999) Mitochondrial DNA variation in Koryaks and Itel'men: population replacement in the Okhotsk Sea-Bering Sea region during the Neolithic. Am J Phys Anthropol 108:1-39.

67. Pliss L, Tambets K, Loogvali EL, Pronina N, Lazdins M, et al. (2006) Mitochondrial DNA portrait of Latvians: towards the understanding of the genetic structure of Baltic-speaking populations. Ann Hum Genet 70: 439-458.

68. Kolman CJ, Sambuughin N, Bermingham E (1996) Mitochondrial DNA analysis of Mongolian populations and implications for the origin of New World founders. Genetics 142:1321-34.

69. Kong QP, Yao YG, Sun C, Bandelt H J, Zhu CL, Zhang YP (2003) Phylogeny of East Asian mitochondrial DNA lineages inferred from complete sequences. Am J Hum Genet 73: 671–676.

70. Yao YG, Kong QP, Wang CY, Zhu CL, Zhang YP (2004) Different matrilineal contributions to genetic structure of ethnic groups in the silk road region in china. Mol Biol Evol 21:2265-80.

71. Tonks et al., unpublished.

72. Osipova et al., unpublished.

73. Bermisheva MA, Kutuev IA, Spitsyn VA, Villems R, Batyrova AZ, et al. (2005) Analysis of mitochondrial DNA variation in the population of Oroks. Genetika 41:78-84.

74. Dupuy BM, Olaisen B (1996) mtDNA sequences in the Norwegian Saami and main populations. In: Carracedo A, Brinkmann B, Bär W, editors. Advances in forensic haemogenetics. Berlin: Springer. pp. 23-25.

75. Opdal SH, Rognum TO, Vege A, Stave AK, Dupuy BM, et al. (1998) Increased number of substitutions in the D-loop of mitochondrial DNA in the sudden infant death syndrome. Acta Paediatr 87: 1039-1044.

76. Passarino G, Cavalleri GL, Lin AA, Cavalli-Sforza LL, Borresen-Dale AL, et al. (2002) Different genetic components in the Norwegian population revealed by the analysis of mtDNA and Y chromosome polymorphisms. Eur J Hum Genet 10: 521-529.

77. Malyarchuk BA, Grzybowski T, Derenko MV, Czarny J, Wozniak M, et al. (2002) Mitochondrial DNA variability in Poles and Russians. Ann Hum Genet 66: 261-283.

78. Pereira L, Cunha C, Amorim A (2004) Predicting sampling saturation of mtDNA haplotypes: an application to an enlarged Portuguese database. Int J Legal Med 118: 132-136.

79. Fedorova SA, Bermisheva MA, Villems R, Maksimova NR, Khusnutdinova EK (2003) Analysis of mitochondrial DNA haplotypes in yakut population. Mol Biol 37:643-53.

80. Pakendorf B, Wiebe V, Tarskaia LA, Spitsyn VA, Soodyall H, et al. (2003) Mitochondrial DNA evidence for admixed origins of central Siberian populations. Am J Phys Anthropol 120:211-24.

81. Zlojutro M, Rubicz R, Devor EJ, Spitsyn VA, Makarov SV, et al. (2006) Genetic structure of the Aleuts and Circumpolar populations based on mitochondrial DNA sequences: a synthesis. Am J Phys Anthropol 129:446-64.

82. Delghandi M, Utsi E, Krauss S (1998) Saami mitochondrial DNA reveals deep maternal lineage clusters. Hum Hered 48:108-14.

83. Tambets K, Rootsi S, Kivisild T, Help H, Serk P, et al. (2004) The Western and Eastern roots of the Saami--the story of genetic "outliers" told by mitochondrial DNA and Y chromosomes. Am J Hum Genet 74: 661-682.

84. Abu-Amero KK, Larruga JM, Cabrera VM, Gonzalez AM (2008) Mitochondrial DNA structure in the Arabian Peninsula. BMC Evol Biol 8: 45.

85. Koledova et al., unpublished.

86. Zupanic Pajnic I, Balazic J, Komel R (2004) Sequence polymorphism of the mitochondrial DNA control region in the Slovenian population. Int J Legal Med 118:1-4.

87. Keyser-Tracqui C, Crubézy E, Ludes B (2003) Nuclear and mitochondrial DNA analysis of a 2,000-year-old necropolis in the Egyin Gol Valley of Mongolia. Am J Hum Genet 73:247-260.

88. Bramanti B, Thomas M, Haak W, Unterlaender M, Jores P, et al. (2009) Genetic discontinuity between local hunter-gatherers and central Europe's first farmers. Science 326:137-140.

89. Krause J, Briggs A, Kircher M, Maricic T, Zwyns N, et al. (2010) A complete mtDNA genome of an early modern human from Kostenki, Russia. Curr Biol 20:231-236.

90. Lalueza-Fox C, Sampietro M, Gilbert M, Castri L, Facchini F, et al. (2004) Unravelling migrations in the steppe: mitochondrial DNA sequences from ancient central Asians. Proc Biol Sci 271:941-947.

91. Keyser C, Bouakaze C, Crubézy E, Nikolaev V, Montagnon D, et al. (2009) Ancient DNA provides new insights into the history of south Siberian Kurgan people. Hum Genet 126:395-410.

92. Haak W, Balanovsky O, Sanchez JJ, Koshel S, Zaporozhchenko V, et al. (2010) Ancient DNA from European early Neolithic farmers reveals their near eastern affinities. PLoS Biol 8 e1000536.

93. Mooder KP, Weber AW, Bamforth FJ, Lieverse AR, Schurr TG, et al. (2005) Matrilineal affinities and prehistoric Siberian mortuary practices: a case study from Neolithic Lake Baikal. JAS 32:619–634.

94. Malmström H, Gilbert M, Thomas M, Brandström M, Storå J, et al. (2009) Ancient DNA reveals lack of continuity between neolithic hunter-gatherers and contemporary Scandinavians. Curr Biol 19:1758-1762.

95. Sampietro M, Lao O, Caramelli D, Lari M, Pou R, et al. (2007) Palaeogenetic evidence supports a dual model of Neolithic spreading into Europe. Proc Biol Sci 274:2161-2167.

96. Mooder K, Schurr T, Bamforth F, Bazaliiski V, Savel'ev N (2006) Population affinities of Neolithic Siberians: a snapshot from prehistoric Lake Baikal. Am J Phys Anthropol 129:349-361.
